# Innovative Analytical Approaches for Food Pesticide Residue Detection: Towards One Health-Oriented Risk Monitoring

**DOI:** 10.3390/jox15050151

**Published:** 2025-09-16

**Authors:** Alexandra Andreea Botnaru, Ancuta Lupu, Paula Cristina Morariu, Alin Horatiu Nedelcu, Branco Adrian Morariu, Maria Luisa Di Gioia, Vasile Valeriu Lupu, Oana Maria Dragostin, Ioana-Cezara Caba, Emil Anton, Madalina Vieriu, Ionela Daniela Morariu

**Affiliations:** 1 Department of Environmental and Food Chemistry, Faculty of Pharmacy, Grigore T. Popa University of Medicine and Pharmacy, 700115 Iasi, Romania; botnaru.alexandra@yahoo.com (A.A.B.); ionela.morariu@umfiasi.ro (I.D.M.); 2 Department of Pediatrics, Faculty of General Medicine, Grigore T. Popa University of Medicine and Pharmacy, 700115 Iasi, Romania; ancuta.ignat1@umfiasi.ro (A.L.); vasile.lupu@umfiasi.ro (V.V.L.); 3 Department of Internal Medicine, Faculty of General Medicine, Grigore T. Popa University of Medicine and Pharmacy, 700115 Iasi, Romania; 4 Department of Morpho-Functional Science I, Faculty of General Medicine, Grigore T. Popa University of Medicine and Pharmacy, 700115 Iasi, Romania; alin.nedelcu@umfiasi.ro; 5 Faculty of General Medicine, Grigore T. Popa University of Medicine and Pharmacy, 700115 Iasi, Romania; morariubranco@gmail.com (B.A.M.); emil.anton@umfiasi.ro (E.A.); 6 Dipartimento di Farmacia, Salute e Scienze della Nutrizione, Università della Calabria, Arcavacata di Rende, 87036 Cosenza, Italy; ml.digioia@unical.it; 7 Research Centre in the Medical-Pharmaceutical Field, Faculty of Medicine and Pharmacy, “Dunarea de Jos” University of Galati, 800010 Galati, Romania; oana.dragostin@ugal.ro; 8 Department of Toxicology, Faculty of Pharmacy, Grigore T. Popa University of Medicine and Pharmacy, 700115 Iasi, Romania; ioana-cezara.caba@umfiasi.ro; 9 Department of Analytical Chemistry, Faculty of Pharmacy, Grigore T. Popa University of Medicine and Pharmacy, 700115 Iasi, Romania; madalina.vieriu@umfiasi.ro

**Keywords:** pesticide residues, monitoring, food safety, chromatography, sample pretreatment, analytical methods

## Abstract

The increasing use of pesticides in agricultural products raises concerns over food safety. Furthermore, uncontrolled pesticide usage on food products can lead to residual levels that exceed the maximum residue limits (MRLs) and are potentially harmful to human health. Long-term consumption of food contaminated with pesticides can contribute to the buildup of toxic substances in the body, which has negative health effects. Advanced analytical techniques are essential to ensure the accurate and effective monitoring of pesticide residues. To ensure adherence to legal requirements, it is essential to employ rapid and accurate methods for detecting these contaminants. This review outlines current advancements (2020–2025) in the assessment of pesticide residues in diverse food matrices, including sample preparation procedures and detection methods. This review provides a standardized comparative analysis of analytical methods for detecting pesticide residues, emphasizing their advantages and limitations, sensitivity, costs, and applicability to complex food matrices, while evaluating its findings through the One Health approach, linking residue evaluation to cumulative exposure and regulatory standards. This study provides practical guidelines for laboratories and regulators while delineating research requirements for more environmentally friendly, rapid, and sensitive residue analysis in accordance with One Health-oriented risk assessment.

## 1. Introduction

Food safety concerns are escalating, posing significant risks to human health. Food contaminants, including heavy metal ions, foodborne viruses, pesticides, mycotoxins, and antibiotics, have emerged as insidious threats to human life and health via dietary consumption and bioaccumulation [1,2,3].

The extensive use of pesticides has contaminated food, feed, water, air, soil, and other sources, posing serious risks to food safety and public health [4]. Approximately 0.1% of the applied pesticide reaches its intended target, while the remaining amount turns into a pollutant in soil and the ecosystem, thus compromising future food sources [5].

Pesticides, owing to their high accumulative nature and persistence, mitigate crop loss and enhance production, which is highly beneficial for farmers. Data indicate that rice production, which sustains almost 50% of the population, has tripled due to pesticide use, while wheat production has risen by approximately 160%. Pesticides allow farmers to cultivate a greater yield on reduced land, thus contributing to the mitigation of deforestation. However, given that the majority of pesticides are toxic, we cannot overlook their detrimental consequences [6].

One Health is characterized as a solid, integrative strategy that aims to sustainably harmonize and enhance the health of humans, animals, and ecosystems. It acknowledges the interconnectedness and interdependence of human health, domestic and wild animal health, plant health, and the broader ecosystem [7]. Toxic chemicals may be identified in air, water, soil, vegetation, food, and animal feed. These residues infiltrate flora and fauna, accumulating in both people and animals through the food chain. They endanger our lives and affect our overall well-being while also annihilating beneficial organisms within the ecosystem. Researchers disclosed that most residual pesticides found in soil, agricultural goods, water, and sediment, including illegal compounds still employed for various purposes, have been recognized as potentially detrimental to ecosystems and human health [8]. The alterations in soil microbial processes, soil characteristics, and enzymatic activity due to pesticide applications are significant elements that considerably influence soil production. Disruptions in microbial community composition may result in significant alterations in the cycling of essential nutrients and metals and their consequent absorption by plants. Recent discussions in both scientific and public forums have notably focused on the detrimental effects of conventional pest control practices, especially the extensive use of neonicotinoid pesticides, on key insect pollinators like bees [9,10]. Moreover, socioeconomic factors affect the exposure of at-risk people to pesticide residues through food, housing, and occupational environments, as well as their capacity to obtain safer alternatives. The One Health concept underscores the inseparable connection among human, animal, and environmental health.

Regardless of progress in organic agriculture, current studies have shown that several food products across various regions of the globe have been contaminated by pesticides [11]. An estimated 385 million cases of accidental acute pesticide poisoning occur annually, with approximately 11,000 deaths from these cases. Approximately 44% of farmers worldwide—roughly 860 million people—suffer from pesticide poisoning each year. Moreover, 20,000 people in developing nations perish from pesticide-contaminated food each year [12]. Studies indicate that several pesticides impact the neurological system, elevating the risk of neurodegenerative disorders. Exposure to pesticides is associated with various health effects, ranging from mild symptoms such as nausea and headache to more severe conditions, including endocrine disruption, cardiovascular disease, cancer, diabetes, congenital anomalies, compromised immune and reproductive systems, Alzheimer’s disease, and Parkinson’s disease [8,13]. Neurodevelopmental toxicity is particularly concerning, as symptoms may present gently and with a delayed beginning following early-life exposure, highlighting the considerable neurotoxic potential of pesticide residues and underscoring the necessity for their thorough consideration in food safety evaluations [14]. Therefore, the potential toxicity of pesticides is a public health issue since it is applicable in various environments, including agricultural areas, roadways, and residential, educational, and leisure institutions [15].

Various techniques have been employed for the quantification of pesticide residue analysis. Analytical technique development consists of two primary steps, which are sample preparation (including matrix pretreatment) and analytical determination of target chemicals [16]. Sample preparation is the most critical stage before instrumental analysis. The progress of extraction methods has resulted in improvements in analytical processes, reducing the complexity of sample treatment while boosting the accuracy and precision of analysis [17]. The analytes must be separated from complex matrices using appropriate extraction, cleaning, and/or preconcentration techniques [18]. Extraction can be accomplished utilizing a solid, liquid, gas, or supercritical fluid as an extractant, contingent upon the following five fundamental chemical properties: hydrophobicity, vapor pressure, solubility, molecular weight, and acid dissociation [19]. Microextraction is garnering more interest due to its sensitivity, simplicity, and efficiency in analyzing complicated matrices. It may be utilized for a wide array of structural configurations and polarity of compounds. Filtration and centrifugation may occasionally be circumvented, using only minimal quantities of organic solvents [20]. Achieving the complete separation of analytes from other sample components through flawless sample preparation is highly challenging and unfeasible in all but a select few situations [21].

Instrumental analysis and rapid detection methods are two of the currently described approaches for detecting pesticide residue. Various factors have contributed to the rapid development in the complexity of analytical equipment over the last few decades. Whereas, in the 1970s, a chromatogram was directly printed on thermal paper, yielding minimal information, today a chromatograph connected to a high-resolution mass spectrometer may gather up to 20 spectra/second. Furthermore, as instrument prices decrease, mass spectrometers have gained popularity in both academia and industry [22]. Traditional techniques used in instrumental analysis include gas chromatography (GC), high-performance liquid chromatography (HPLC), and mass spectrometry (MS) [23]. Analytical methods have recently evolved from single to multiple residue analysis, owing to the use of highly selective analytical methods like liquid chromatography tandem mass spectrometry (LC-MS/MS) and gas chromatography tandem mass spectrometry (GC-MS/MS). Such new technologies have substantially simplified analytical processes, allowing for the identification of pesticide residues at trace levels with exceptional accuracy and precision.

Multiresidue techniques enable the analysis of a large number of chemicals with excellent recovery rates while also addressing possible sample interference. Regarding rapid detection techniques, biosensors are the most promising currently employed. A possible approach is the use of sensors employing diverse transduction principles, including fluorescence, colorimetry, and electrochemistry. Biosensors will play a crucial role in detecting pesticide residues in fruits and vegetables [24]. To ensure food safety and environmental sustainability, biosensors have the potential to either replace or enhance conventional analytical methods for monitoring pesticides in agricultural products [25]. As analytical platforms, they may be handled, offering on-site data, which are particularly advantageous for identifying pesticide residues in imported food at control points (border inspections, field testing) [26].

Prior reviews of pesticide detection have mostly focused on methods or matrices. Expanding upon this literature, we present a standardized, decision-oriented comparison of analytical workflows (environmental sustainability, cost, throughput, sensitivity, and efficacy in complex food matrices) while explicitly adopting a One Health perspective by contextualizing recent advancements within regulatory frameworks and linking food residues to environmental and biomonitoring evidence.

## 2. Pesticide Types, Characteristics, and Regulatory Framework

**Figure 1:**
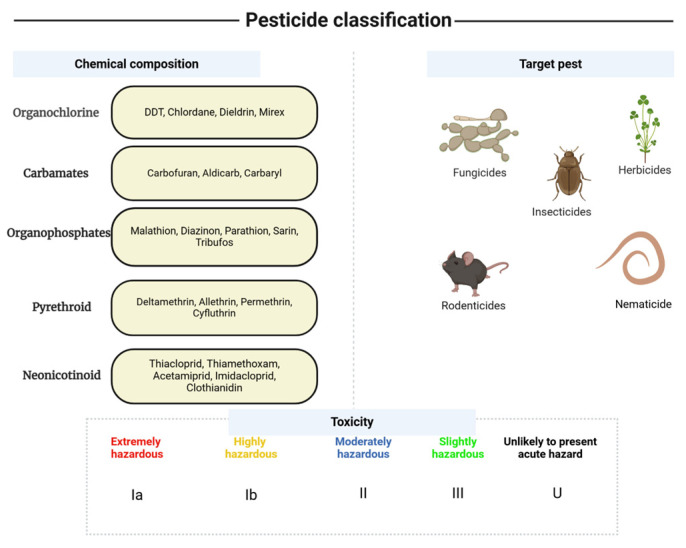
Pesticide classification by chemical structure, targeted pest species, and toxicity (created with BioRender.com).

Organochlorine pesticides (OCPs), carbamates, organophosphates (OPPs), pyrethroids, and neonicotinoids are among the most commonly used types of pesticides in agriculture and pest management, depending on their chemical composition. The main classification criteria for pesticides are their chemical structure and their mode of action or mode of entry, which describe how the pesticide controls or gets rid of the target pest (Figure 1). Every type of pesticide has unique properties and different ways of action [12,27,28,29,30].

### 2.1. OCPs

**Figure 2:**
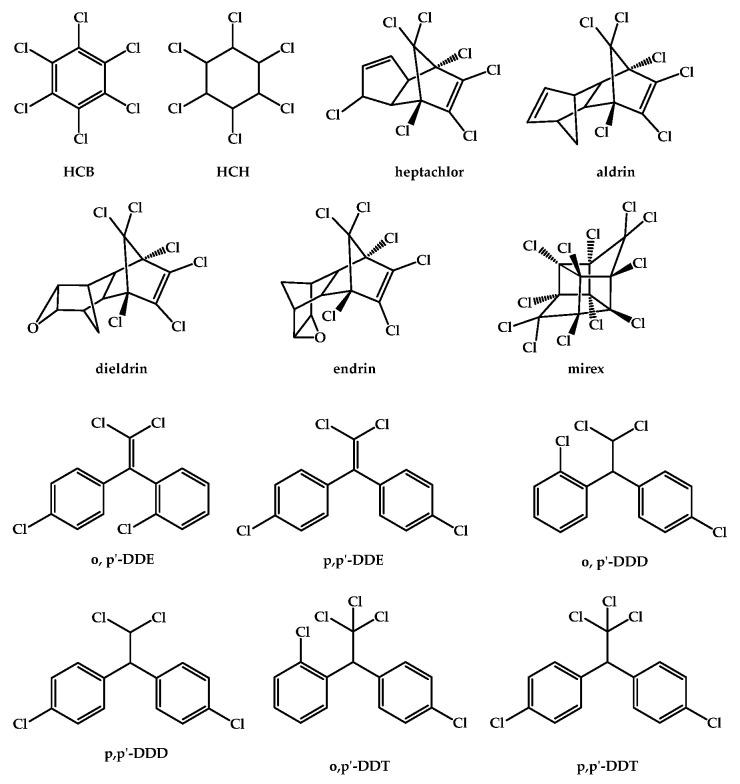
Chemical structure of OCPs. Modified and adapted after Adeyinka et al. [40].

OCPs are chlorinated hydrocarbons that are extensively utilized in crops for pest control [31]. OCPs are classified as high-persistence organic pollutants in the environment. Formerly used to combat typhus and malaria, these insecticides are currently prohibited in a large number of countries [32]. Due to their persistence in the environment, ability to bioaccumulate in the food chain, and ability to accumulate in human adipose and other tissues, the extended use of organochlorines such as p,p′-dichlorodiphenyltrichloroethane (DDT), its metabolite dichlorodiphenyldichloroethylene (DDE), heptachlor epoxide, hexachlorobenzene, β-hexachlorocyclohexane, oxychlordane, and trans-nonachlor is particularly relevant to human health [33]. The chemical structure for selected OCPs is represented in Figure 2 [34].

From a toxicological perspective, OCPs are primarily linked to chronic health risks. Experimental studies provide evidence that these chemicals possess carcinogenic potential by interacting with steroid signaling pathways and promoting the growth of breast cancer cells that exhibit hormone receptors [35]. Freire et al. distinctly identified a significant correlation between blood levels of certain organochlorine pesticides and elevated total T3 in children, indicating possible thyroid-disrupting effects [36]. The GerES V research indicates a sustained reduction in plasma concentrations of organochlorine pesticides in German children and adolescents, while these enduring compounds remain detectable. Despite a minimal overall health risk, geographical disparities and food-related exposure pathways, including fish intake and breastfeeding, underscore the necessity for continuous monitoring [37].

Due to their significant persistence, bioaccumulation, and mechanisms of endocrine disruption and carcinogenicity, organochlorine pesticides (OCPs) constitute one of the most hazardous pesticides, with health effects mostly due to chronic exposure rather than acute toxicity. Moreover, soil residues continue to be a source of environmental re-emission even decades post phase-out [30,38,39].

### 2.2. OPPs

**Figure 3:**
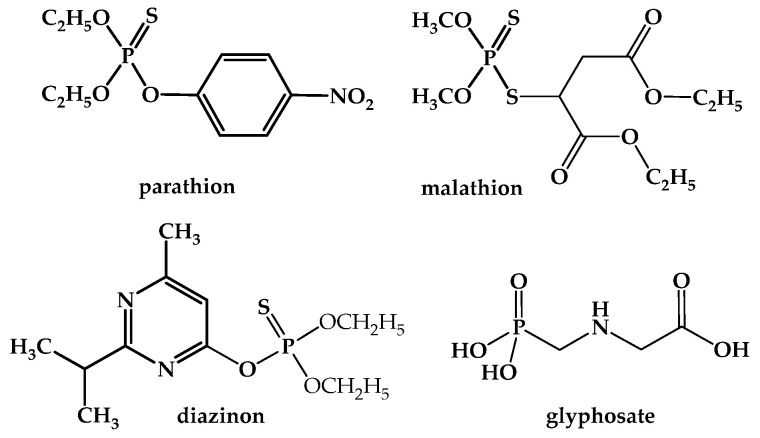
Chemical structures of commonly used OPPs. Modified and adapted after Mdeni et al. [42].

The vast quantity of OPPs that persist in food, drinking water, soil, and air can enter the human body by oral, skin, inhalation, or ocular contact, posing health risks. Chemical structures of widely used organophosphate insecticides are presented in Figure 3 [41].

Only 10–15% of OPPs used in the field are effective, and a large number of residual OPPs are released into the aquatic environment because the majority of them are very soluble. This highlights a major worry about the high toxicity of parent OPPs and their metabolites [43]. Parathion, endogenous phosphorus, malathion, dimethoate, and trichlorfon are the most prevalent organophosphates employed as insecticides in the management of plant pests [44]. Ethyl parathion is one of the most extensively utilized pesticides in both agricultural and non-agricultural sectors. Conversely, according to the EPA, ethyl parathion is documented as one of the most dangerous substances in its registry [45].

### 2.3. Carbamates

**Figure 4:**
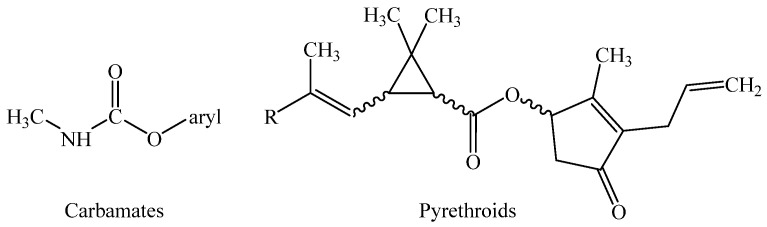
General structure of carbamate and pyrethroid pesticides. Modified and adapted after Hassaan and El Nemr and Tomasevic et al. [30,49].

Carbamates are a large class of compounds that include carbamic acid esters and thioesters (Figure 4). They are usually soluble in water and polar organic solvents and are available commercially as wet powders, dust granules, and emulsion concentrates. Soil microbes typically degrade these chemicals within three to five weeks [38].

Carbamates are insecticides that inhibit the enzyme AChE, allowing acetylcholine to accumulate in the nervous system. This may cause symptoms like sweating, excessive salivation, clouded vision, and, in extreme instances, paralysis and breathing difficulties [12,46].

These chemicals affect male fertility by disrupting hormonal regulation and influencing sperm production [12]. Exposure to carbamates during infancy has been associated with negative health outcomes and has garnered significant attention [47]. Furthermore, several carbamates are believed to possess carcinogenic and mutagenic properties [48].

### 2.4. Pyrethroids

Pyrethroids are natural insecticides obtained from the pyrethrum extracts of chrysanthemum flowers, namely pyrethrin, found in Kenya [30]. Synthetic chemical compounds (Figure 4) called pyrethroids are well-known for their ability to effectively control a variety of insect pests. Their strong insecticidal qualities make them useful in pest control and agriculture [12,26,38,50].

Pyrethroids may lead to contamination of the food chain, resulting in the bioaccumulation of these pesticides in animal-derived products, including meat, fish, milk, and honey [51]. Even while pyrethroids are usually less hazardous to people than OPPs, there are still risks associated with them, particularly when exposure is extended. Pediatric patients are a vulnerable demographic subjected to pesticide exposure through multiple pathways [15]. Recent epidemiological and longitudinal research demonstrates that prenatal exposure to pyrethroid pesticides correlates with a heightened risk of autism, developmental delays, and other developmental abnormalities in children [14].

### 2.5. Neonicotinoids

**Figure 5:**
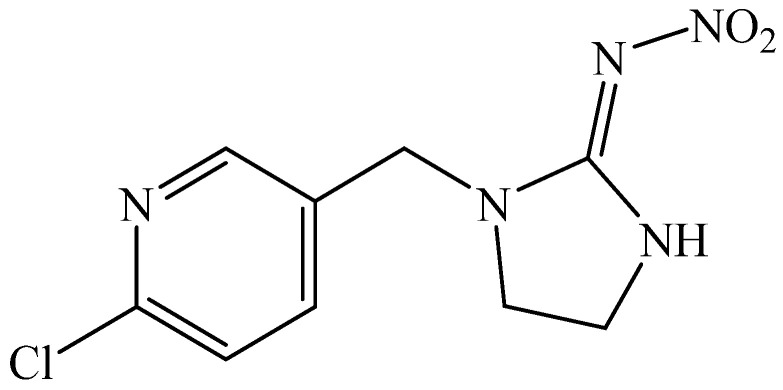
Chemical structure of imidacloprid. Modified and adapted after Yari et al. [55].

Recently, neonicotinoids have been the fastest-growing group of insecticides in modern agricultural protection [52]. These insecticides have characteristics comparable to those of nicotine, although they pose less of a risk to humans. Neonicotinoids and nicotine are connected chemically. After the discovery of imidacloprid, various analogs, including the 6-chloro-3-pyridylmethyl moiety, were generated, including acetamiprid, nitenpyram, and thiacloprid. Figure 5 shows the structure of imidacloprid [29,53,54].

Neonicotinoids are effectively utilized in agriculture for crops like maize, cotton, oilseed rape, sunflower, and sugarcane, owing to their superior solubility, chemical characteristics, and selective control, which facilitate their distribution in plants through xylem and phloem transport mechanisms [56].

From a toxicological–mechanistic perspective, neonicotinoids act as agonists of nicotinic acetylcholine receptors, resulting in acute or subacute neurotoxic effects in target insects and, following sufficient exposure, in non-target species [50,52]. Their ecotoxicological impact is thoroughly documented as follows: non-target species, particularly pollinators and aquatic invertebrates, are exposed through various pathways (seed coatings, foliar sprays, contaminated dust, and soils), with field data revealing minimal plant uptake from seed treatments, resulting in the majority of the active ingredient remaining in the soil or as sowing dust. These characteristics, along with environmental persistence, can extend ecosystem-level exposures [38,57].

The environmental outcome of neonicotinoids is influenced by several parameters, such as their water solubility, adsorption to soil and sediment, input–removal dynamics, and existing abiotic conditions. Degradation processes ultimately determine their permanence, serving as the primary mechanism for removal from polluted environments. Neonicotinoids undergo degradation through a combination of abiotic transformations, including photolysis and hydrolysis, and biotic reactions facilitated by microorganisms and plants [58]. Specifically, photochemical degradation under direct sunlight generates several intermediate metabolites, some of which possess biological activity and may lead to prolonged ecotoxicological impacts in aquatic and soil environments [59].

Regarding humans, exposure data—particularly for children—remain limited, but epidemiological and longitudinal findings suggest neurodevelopmental endpoints associated with postnatal exposure to compounds such as clothianidin and imidacloprid [60,61].

### 2.6. Regulatory Framework

To protect human health and facilitate international commerce, the European Union and the Codex Alimentarius Commission established MRLs for pesticide residues in food products. The European Food Safety Authority (EFSA) performs thorough risk evaluations of chemical residues in food and feed within the European Union, collaborating with state agencies to deliver transparent scientific judgments that inform policy-making [62]. In the United States, regulatory authority is distributed among the EPA (Environmental Protection Agency), USDA (Department of Agriculture), and FDA (Food and Drug Administration), with the EPA responsible for pesticide registration, setting maximum residue levels (MRLs), and assessing environmental effects. The US EPA registers pesticides, establishes MRLs for raw agricultural commodities, and investigates the environmental impact of residues. The European Union has established an extensive legal framework that delineates regulations for the authorization of active chemicals under Regulation 1107/2009, their use in plant protection products, and their allowable residues in food. The MRLs denote the maximum legally permissible concentration of pesticide residue in or on food, provided that a plant protection product is utilized in compliance with its Good Agricultural Practice (GAP) [63]. The goal is to develop MRL values that decrease pesticide residues in food to the lowest possible and acceptable levels for consumers, following sensible application to preserve crops [14]. MRLs and Health-Based Guidance Values (HBGVs) have separate but complementary roles in risk assessment and food safety regulation. HBGVs, encompassing the Acceptable Daily Intake (ADI) and the Tolerable Upper Intake Level (UL), are scientifically defined parameters that specify the amount of a substance that may be consumed daily during a lifetime without posing a substantial health hazard. These numbers are generally based on toxicological data and are employed to assess total dietary exposure from all sources. Conversely, MRLs are regulatory limits set for the presence of residues from substances such as pesticides or veterinary medications in food products. They aim not to indicate safety directly but to ensure that the presence of such residues in food does not lead to consumer exposures exceeding the pertinent HBGVs. Thus, HBGVs function as toxicological benchmarks, whereas MRLs act as regulatory tools designed to ensure that actual exposure remains below permissible thresholds [64]. The cumulative risk assessment is a significant concern, as the MRLs are established for individual residues, whereas food may be contaminated with several pesticide residues [26].

Exposure modeling is an essential element of dietary risk evaluation. It assesses consumer exposure by integrating residue levels with food consumption data and contrasts the values to toxicological parameters such as the ADI and Acute Reference Dose (ARfD). The following two principal methodologies are employed: long-term (chronic) exposure assessment, which utilizes mean residue concentrations and average consumption to calculate the Estimated Daily Intake (EDI), and short-term (acute) exposure assessment, which assesses high-percentile intakes relative to the Acceptable Risk of Dose (ARfD) through the International Estimated Short-Term Intake (IESTI) model [65,66].

A wide range of programs are being conducted to assess, monitor, and reduce consumer exposure to pesticide residues in the food supply. For example, to safeguard the Brazilian populace from significant hazards linked to pesticide-contaminated food, the Brazilian National Sanitary Agency has implemented a comprehensive monitoring program for pesticide residues in fruits and vegetables since 2001. In 2009, an analysis of 20 varieties of fruits and vegetables revealed that 23.2% had pesticide residues, with 14.3% of the samples surpassing the European Union’s MRLs [67]. Analytical advancements—multiresidue LC/GC-MS/MS with validated LOQ, identification criteria, and extraction efficiency control—directly guide enforceable maximum residue level decisions under Codex/EU/US regulations and facilitate One Health risk assessment by correlating residues across food, water, biota, and human biomonitoring.

## 3. Sample Pretreatment

The common approach for detecting and quantifying pesticide levels in samples is based on chromatographic methods (GC, LC, HPLC, UHPLC, SFC) with different detectors, although the sample preparation procedure is often matrix-specific [68]. Various sample preparation approaches have been proposed to extract pesticide residues from food products; nevertheless, we highlight the most effective and thoroughly investigated methods. Contemporary trends in analytical chemistry emphasize the simplification and miniaturization of the analytical process. The traditional liquid–liquid extraction method is hindered by time consumption, reliance on harmful volatile organic chemicals, and the generation of substantial waste solvent volumes. Consequently, to mitigate these constraints, researchers have devised many microextraction techniques [69].

### 3.1. MAE (Microwave-Assisted Extraction)

MAE is a technique that uses microwave radiation to heat solvents in contact with a sample, facilitating the transfer of analytes from the substrate to the solvent; hence, it provides high sample throughput with minimal solvent usage [70]. This process involves the absorption of electromagnetic waves by solid materials, which converts them into thermal energy. Subsequently, when pressure is exerted on the solid cell wall, it results in cell expansion; as the pressure escalates, the cell fractures, allowing organic pollutants inside the solid cell to leach into the organic solvent [71]. The use of MAE offers several benefits, including a high extraction rate, automation, and the potential for simultaneous sample extractions without interference. In the past few years, the use of microwaves for the extraction of constituents, primarily from plant tissues, has garnered significant study attention [70]. Only thermally stable compounds may be utilized with this technique, which poses the possibility of decomposing temperature-sensitive chemicals, and they must be dissolved in a polar solvent such as water [17,72]. However, its availability can be limited in other laboratories, as it requires an expensive instrument.

Tian et al. adopted this technique to extract mancozeb from fruits and vegetables, requiring just 50 s of pretreatment time. The mean recoveries of mancozeb varied between 81% and 112%. LOD and LOQ were 0.003 and 0.01 mg kg^−1^, respectively [73]. Zondo et al. implemented this method effectively for the quantification of herbicides in maize crops. The recoveries of herbicides in maize ranged from 80% to 98%. The concentrations of herbicides measured varied from 2.7 to 20.4 µg L^−1^ [71].

### 3.2. ASE (Accelerated Solvent Extraction)

ASE is a solid–liquid organic component extraction method that operates at high temperatures (50–200 °C) and pressures (10–15 MPa), combining the advantages of high throughput, automation, and little solvent usage [74]. High temperature and high pressure are required to obtain higher extraction rates owing to lower viscosity and surface tension. However, the process also enhances the diffusion rate and solubility in the matrix [75]. This novel sample extraction technology provides various benefits, including cheap extraction costs, decreased solvent and time consumption, and streamlined extraction processes [74]. As a drawback, ASE entails considerable investment in equipment and maintenance, and the assembly and disassembly of sample extraction cells may provide challenges [76].

Currently, honey is produced in a contaminated environment. Consequently, modern honey contains a variety of chemical residues, including pesticides [77]. Upon analysis, over fifty percent of the samples revealed a combination of pesticides [78]. To separate pesticide residues from organic honey samples, two in-line ASE extraction techniques were devised and evaluated. Florisil and PSA (primary secondary amine) were used as interference retainers. The ASE with in-line clean-up is cost-effective and reduces waste creation compared to conventional procedures; by integrating extraction and clean-up in a single step, the time needed for the analysis is reduced [75]. Zhang et al. revealed that the average recovery of target compounds using ASE was 96%, with the majority of compounds falling within the confidence levels [74].

### 3.3. SPE (Solid Phase Extraction)

SPE techniques have been extensively utilized for the LC analysis of quaternary ammonium pesticide residues. Mixed-mode polymeric SPE cartridges can enhance analyte extraction [79]. Advantages include convenience, cost-effectiveness, simplicity, decreased organic solvent usage, the possibility for multiresidue analysis, and compatibility with different detection methods. This method offers a variety of sorbents with distinct chemical structures, allowing for alternative extraction methods to accept pesticides with variable physicochemical qualities [80].

An examination of analytical efficiency based on cleanup techniques was carried out for Korean cabbage samples. The percentage of pesticides in these food samples that fell within the proper recovery rate range was 94–99% for SPE [81]. Chemical interferences have diminished over time owing to the enhanced selectivity attained by methods such as SPME, SPE, UHPLC, and complete two-dimensional gas chromatography (GC × GC) [21]. Numerous co-eluting chemicals make it challenging to analyze vegetable oils for pesticides. For instance, SPE with an alumina column or a C18 sorbent is often used [68].

### 3.4. dSPE (Dispersive Solid Phase Extraction)

The benefit of dSPE is its requirement for minimal laboratory apparatus, specifically a centrifuge and a vortex. It employs reduced sorbent material, necessitates fewer sample quantities, and provides superior interaction between the sorbent and extract compared to traditional SPE [51]. Multiple techniques have been employed to eradicate co-extracted interference from extracts, including freezing centrifugation, SPE, and dSPE. Nonetheless, except for dSPE, other clean-up methods are labor-intensive and need substantial quantities of solvent. Furthermore, certain clean-up methods may lead to the loss of pesticides due to adsorption onto sorbents or during the concentration of the extraction solution [51].

In order to obtain the removal of co-extracted substances, dSPE using various sorbent types was suggested. The hydrophobic character of the sample is maintained by applying C18, while primary–secondary amine sorbents may be utilized to achieve significant retention of fatty acids, organic acids, pigments, and sugars [68]. Clean-up is a required step for identifying pesticides in food that has a high concentration of polyphenols (apple, tea, and broccoli). In a method previously utilized in a study to enhance the precision of polyphenol quantification, the polyphenols were precipitated using d-SPE and polyvinylpolypyrrolidone as part of the sample pre-treatment step before the sample was diluted to remove the matrix impact [68].

Yun et al. examined 272 pesticide residues in food samples, including brown rice, soybeans, green peppers, mangos, and potatoes. The d-SPE technique was employed for the cleanup step, utilizing MgSO_4_ and PSA sorbents to enhance sample purification [82].

### 3.5. MSPD (Matrix Solid-Phase Dispersion)

MSPD, introduced in 1989, is an adaptation of SPE that employs a sorbent functioning as an abrasive to create a modified aperture in the solid matrix, facilitating extraction [17].

This extraction has several uses as a sample preparation technique for extracting physiologically active chemicals, naturally occurring components, and other substances from complex biological matrices. MSPD requires the direct mechanical amalgamation of a sample using a sorbent mostly composed of silica bonded to octadecyl groups. The method entails the homogenization of a minimal quantity of a sample [83]. Multiresidue approaches like MSPD have been effectively used to address some of the limitations of the traditional solvent extraction of pesticide residues [84]. This approach requires decreased sample quantity and amount of organic solvent, and it may accomplish preparation, extraction, and fractionation in one step [85].

Kemmerich et al. presented a simplified alternative to the traditional MSPD method, known as balls-in-tube matrix solid-phase dispersion (BiT-MSPD). Using steel balls, this method simplifies sample preparation by carrying out every step inside a closed extraction tube. A total of 133 pesticide residues in fruits (apple, peach, pear, and plum) were successfully quantified using UHPLC-MS/MS. BiT-MSPD is a promising method for pesticide residue analysis since it is quicker, easier, and more effective than traditional MSPD [86].

### 3.6. SPME (Solid-Phase Microextraction)

Over three decades ago, Pawliszyn and coworkers developed SPME, a flexible sample preparation technology that is still widely used today. The technology allows samples to be extracted and preconcentrated in a single step [87]. SPME is a sample preparation technique that uses minimal quantities of an extraction phase to extract target analytes from examined sample matrices [88]. Additionally, SPME provides the concept of microextraction, but it outperforms other traditional sample preparation techniques, such as liquid extractions and SPE, proving great enrichment, high simplicity, flexibility, and, in many instances, reusability in a manner that is environmentally friendly [89].

Due to the complexity of food matrices, SPME is often conducted from the headspace. This technique allows the investigation of chemicals in solid matrices without solvents and in a shorter time [68]. SPME can be applied to gaseous, liquid, and solid matrices [90]. However, the matrix effect poses a challenge in achieving accurate quantitative results with SPME.

A primary research focus of SPME is the development of functionalized materials as extraction phases to facilitate the selective extraction of target pollutants. SPME now extensively uses covalent organic frameworks, carbon compounds, and metal–organic frameworks as coatings [91]. Hydrophobicity/hydrophilicity, pH, and matrix chemicals have a role in how effectively the analytes of interest may be sorbed onto the SPME fiber [87]. However, one of the primary limitations of adopting those technologies is acquiring pure and well-characterized materials, including the fact that they are not commercially available, which causes problems for research facilities and industries [90].

According to Agatonovic-Kustrin et al. researches used SPME combined with HPLC to analyze four pesticides applied to strawberry crops. Four SPME fibers were examined in the process of developing their methodology. When the pesticide combination was injected directly into the HPLC, the sensitivity was approximately five times lower than when an SPME fiber was used [88].

An SPME-coupled HPLC approach was investigated for determining trace amounts of dicamba, 2,4-dichlorophenoxyacetic acid (2,4-D), and picloram residues simultaneously from complex food samples. The SPME system was developed by immobilizing flavonoid moiety-incorporated carbon dots, and as an effective extractant, functional group-incorporated carbon dots demonstrated desirable sensitivity and selectivity to carboxyl-containing aromatic herbicides [92].

Herbicides containing triazine are frequently used on crops to fight weeds. Agatonovic-Kustrin et al. presented an SPME HPLC-MS technique for analyzing seven triazine herbicides [88].

### 3.7. SFE (Supercritical Fluid Extraction)

SFE employs a supercritical fluid, an element that displays characteristics of both a gas and a liquid when above its critical point [93]. Due to its cost-effectiveness and non-toxicity, CO_2_ is commonly employed as the supercritical solvent in supercritical fluid extraction procedures [94]. SFE provides the capability to modulate the solvent power of CO_2_ by altering the density of the supercritical solvent, allowing for more selectivity in the process compared to liquid CO_2_ [95].

In comparison to conventional extraction techniques, SFE has advantages like reduced organic solvent usage, enhanced extract selectivity, and abbreviated processing durations. The closed system of SFE restricts contamination and inhibits oxidation and degradation of the extracted substances by excluding air and light [93]. The primary drawback of CO_2_ is its non-polar nature, which results in supercritical carbon dioxide primarily extracting non-polar or low-polar molecules. In addition to the necessity for high-purity CO_2_ or other supercritical solvents, the demand for substantial capital expenditure is a key issue constraining the application of this extraction method [96].

### 3.8. QuEChERS

The QuEChERS approach is a quick, easy, cheap, effective, rugged, and safe alternative to traditional sample preparation for the multiresidue analysis of diverse chemicals [97]. The QuEChERS method was first presented as a liquid–liquid distribution technique that included combining MgSO_4_ and NaCl to separate an aqueous solution from an organic one [82]. This approach is versatile and may be adapted depending on the characteristics of the analytes, the composition of the sample matrix, and the available analytical equipment [98]. The QuEChERS method may be used for a broad range of analytes, including polar, semi-polar, and non-polar pesticide pollutants in various food matrices [18]. One of the most significant advantages of QuEChERS is its minimal equipment needs and cost when compared to other extraction methods. The application of QuEChERS for sample preparation in pesticide analysis has broadened to encompass fatty meals, including products of animal origin [99].

The QuEChERS technique shows great promise for the study of various types of pesticides in foods [100]. Because of how it can be coupled to chromatographic equipment (gas and liquid chromatography), it provides a broad analytical scope with a wide range of sensitivity and selectivity [98]. It is the preferred approach for food analysis since it combines many processes and increases the number of pesticides recovered compared to earlier, more time-consuming extraction procedures [100].

### 3.9. HF-LPME (Hollow Fiber Liquid-Phase Microextraction)

A novel LPME configuration known as HF-LPME has garnered significant research interest due to its ability to deliver a high analyte preconcentration factor for certain analytes. Moreover, it exhibits exceptional cleaning efficiency, since the HF functions effectively as a filter [101]. The technique must be repeatable and deliver an adequate signal to achieve high sensitivity [102]. This method was primarily developed for the extraction of ionic or polar analytes, including acids, bases, and metals [103]. The efficacy of an HF-LPME approach is often characterized by a pre-concentration factor (PF) or enrichment factor (EF) instead of extraction efficiency [103].

The HF-LPME techniques can be executed in two modes: three-phase and two-phase HF-LPME methods. The initial technique involves an aqueous–organic–aqueous system where the immobilized organic solvent or supported liquid membrane is subjected to two aqueous phases of the sample solution, with the aqueous acceptor phase situated within the hollow fiber. In the two-phase mode, a water-immiscible solvent is infused into the lumen of the HF, functioning as an acceptor phase [104]. Both two-phase and three-phase HF-LPME have been employed for the extraction of environmental pollutants and toxins, with this section focusing on recent advancements and uses of the approach in this domain [105].

HF-LPME is an eco-friendly sample preparation method necessitating just a few microliters of organic solvent per sample. HF-LPME facilitates significant enrichment and superior sample purification from biological and environmental components [106]. Limited publications exist about HF-LPME of foods and drinks. This is unexpected, considering that the approach is very appropriate for the extraction of pesticides. Nonetheless, the published articles unequivocally illustrate the potential of HF-LPME [106].

### 3.10. DLLME (Dispersive Liquid–Liquid Microextraction)

DLLME was developed in 2006 as a fast and inexpensive microextraction method with excellent analyte recovery and enrichment factors, which is particularly appropriate for extracting partially hydrophobic compounds from aqueous solutions [18].

DLLME is a tri-solvent system wherein the disperser solvent acts as an intermediary between the sample solution and the extractant, owing to its solubility and miscibility with both components. For an effective DLLME procedure, the volume of the disperser solvent must exceed that of the extractant to provide sufficient distribution throughout the sample solution. However, DLLME is less adaptable than SPE because of the restricted solvent selection criteria and the fact that it is a time-consuming method involving various stages [22].

Petrarca et al. established a more accurate and environmentally friendly approach for determining pesticide residues in soybeans. They coupled a traditional extraction approach with a DLLME stage, using a deep eutectic solvent—camphor–hexanoic acid (1:1 molar ratio)—for microextraction [107]. In a similar study, pesticides were extracted from tropical fruits, and the analytes were preconcentrated using DLLME prior to trace-level determination [18].

Zgoła-Grześkowiak et al. validated a rapid extraction technique for detecting various pollutants in chicken liver samples using LC-MS/MS. This was the first report of a DLLME approach for simultaneously determining numerous contaminants in biological chicken matrices, including aflatoxin B1, pesticides, fluoroquinolones, sulphonamides, and anthelmintics [83].

### 3.11. SDME (Single Drop Microextraction)

SDME was initially documented in the literature by Liu and Dasgupta in 1995, when a single drop of liquid served as an interface to capture diffusible gas components. Minimal amounts (<10 mL) of extractants are utilized in SDME processes, rendering it a very environmentally friendly analytical approach [19]. To enhance the robustness of the SDME approach, many advancements have been implemented regarding the solvent utilized as an extractant and the design of the equipment for generating or containing the micro-drop [19]. Extraction efficiency may be enhanced by selecting a suitable solvent, minimizing the volume ratio of acceptor micro-droplets to the sample, optimizing the conditions and pH of the donor and receiver stages, and employing auxiliary reagents during the extraction phase to capture the analyte [108].

This extraction technique is recognized as a simple and efficient technique for sample preparation, adeptly extracting and concentrating diverse analytes from complex sample matrices [20]. SDME was employed with GC-MS to analyze OCPs in vegetable samples [109].

### 3.12. CSDF-ME (Continuous Sample Drop Flow Microextraction)

CSDF-ME was devised to miniaturize and address some constraints of the continuous-flow microextraction (CFME) approach [110]. This method involves the extraction of the analyte by passing droplets of the aqueous sample solution through several microliters of an organic solvent that is immiscible with water [111]. The primary benefits of this approach are minimal utilization of extraction solvents, ease of implementation, elevated enrichment factors, and significant stability of the extraction solvents [112]. This method has been demonstrated to be repeatable and more efficient than alternative LPME (liquid-phase microextraction) methods. This improvement is attributable to a reduction in the steps of the sample preparation techniques [113].

A comparative overview of the extraction techniques discussed in this section, highlighting their main advantages, limitations, and practical considerations, is presented in Table 1.

Extraction efficiency may be assessed by many metrics, including recovery rate (RR), enhancement factor (EF), and extraction recovery (ER). ER assesses the efficacy of an extraction procedure. Despite low extraction efficiency, a high enrichment factor renders the approach viable for real sample analysis [102]. The efficiency of the mentioned extraction and clean-up techniques may be examined in Table 2 by a comparative analysis based on three analytical specifications.

## 4. Detection Methods

Pesticide detection methods differ based on laboratory facilities, ensuring accessibility and relevance to varying resource levels. Laboratories with constrained infrastructure utilize quick and lab immunochemical assays, such as test strips or basic ELISA kits, which deliver prompt outcomes at a minimal cost, although with reduced sensitivity and specificity. Laboratories with moderate resources can employ improved ELISA and other immunochemical techniques, allowing for more precise detection and quantification of many chemicals concurrently, although they still need fundamental equipment and skilled workers. In adequately equipped laboratories, chromatography and mass spectrometry techniques, such as LC-MS/MS or GC-MS, provide elevated sensitivity and specificity, facilitating multi-residue analysis and advanced standardization; nonetheless, they entail significant expenses and need expert people. The selection of a detection technique is contingent upon resource availability, existing infrastructure, and testing objectives, hence assuring an extensive and adaptable application [132,133].

**Figure 6:**
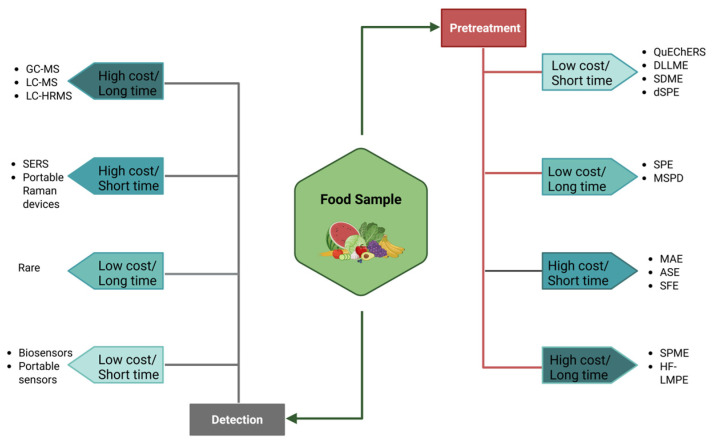
Overview of food sample pretreatment and detection methods classified according to cost-efficiency and time requirements (created with Biorender.com).

A comprehensive schematic illustration of the pretreatment and detection methodologies employed in pesticide residue analysis, categorized by cost-effectiveness and time requirements, is presented to facilitate comparison between rapid, economical techniques and more costly, time-intensive laboratory-based methods (Figure 6).

### 4.1. Chromatography Techniques

Affordable detection approaches are essential for enhancing pesticide residue monitoring in low- and middle-income countries. In several areas, access to high-performance equipment like LC–MS/MS is constrained by substantial acquisition and maintenance costs, the necessity for a specialist, and infrastructural needs. Consequently, cost-effective and accessible screening instruments provide an effective first level of surveillance. In addition to ELISA-based screening, several countries have also implemented monitoring programs and pesticide residue studies employing chromatographic techniques for confirmatory analysis (tropical fresh fruit in Brazil, fresh products in South Africa, and dairy milk in India) [134,135,136].

Instrumental parameters including sensitivity, selectivity, linearity, precision, accuracy, and calibration can influence the accuracy and precision of analyte concentration. Optimizing these settings is essential for achieving the most accurate results. The selection of the most suitable instrument-optimized method for quantifying analyte concentration is contingent upon several parameters, including the analyte’s characteristics, sample concentration, sample matrix, solvent purity, needed sensitivity, and the desired accuracy and precision levels [137].

Alongside conventional GC-MS library matching methods, significant advancements in machine learning (ML) methodologies have emerged, facilitating the enhanced qualitative identification of chemicals and more reliable measurement of concentrations. These algorithms can identify intricate patterns in spectra, minimizing errors linked to analogous chemicals, and they can swiftly adjust to new data sets, hence providing enhanced flexibility and performance relative to traditional methods [138]. Artificial intelligence (AI) and ML have had significant progress and are swiftly gaining prominence in several predictive domains due to their capabilities, precision, and rapidity [139]. In pesticide-residue analysis, AI/ML-assisted spectrum interpretation is anticipated to enhance robustness, mitigate analyst bias, and facilitate high-throughput monitoring in intricate food matrices.

HPLC has become a key tool in pesticide analysis as the agricultural sector seeks to produce more polar pesticides that have reduced volatility and are easily degradable [22]. However, a notable drawback of HPLC is its high solvent consumption. Other chromatographic procedures need multistep sample preparation, which is laborious and time-consuming. Furthermore, these instruments are expensive and have a significant carbon footprint, rendering them unsuitable as available sensors due to calibration complications [140]. The increasing application of highly polar and ionic herbicides such as glyphosate and 2,4-D, as well as the development of HPLC coupled with MS, has made this approach more widely used in pesticide analysis. However, GC remains the usual technique for analyzing semi-volatile and non-polar pesticides [22].

To improve selectivity, MS was subsequently integrated with GC [141]. Various studies have demonstrated the efficacy of LC-MS/MS and GC-MS, respectively [142,143,144,145]. GC, GC-MS, and GC-MS/MS are frequently employed due to their superior separation efficiency, selectivity, and identification capabilities of mass spectrometry [17]. Moreover, the diverse sensitive detectors integrated with GC, including the nitrogen phosphorus detector (NPD), flame ionization detector (FID), flame photometric detector (FPD), and electron capture detector (ECD), have enhanced the detection and quantification of pesticides. The ECD is particularly effective for organochlorine pesticides, the NPD for organophosphorus and nitrogenated pesticides, and the FPD for sulfur and phosphorus pesticides [17]. The utilization of a gas chromatography flame photometric detector (GC-FPD) is very successful in identifying organophosphorus pesticides, providing enhanced accuracy and superior experimental results [146]. Despite the initial lack of uniform nomenclature among chromatographers, matrix effects have inevitably influenced several GC investigations [21].

LC–MS is extensively utilized, as it enables the simultaneous analysis of several pesticides, their metabolites, and degradation products in a single run. The primary drawback of LC–MS is the high cost of the instrument, together with its operational and maintenance expenses [141]. Numerous compounds in plant-based foods that are infrequently examined or challenging to identify, such as highly polar, non-volatile, or thermally unstable pesticides, can be swiftly and efficiently detected using the LC-MS technique, including those unsuitable for GC. Advancements in LC-MS/MS now enable the detection of pesticide residues in intricate matrices such as fruits, vegetables, cereals, and animal-derived products [17]. Its primary advantages are very high sensitivity, low LOD, and excellent selectivity [141]. Nonetheless, these techniques exhibit the following issues: The extraction and purification process entails numerous analytical procedures that are challenging and time-intensive. Recovery rates are often low and inconsistent, and the sample preparation or analysis lacks cost-effectiveness. A significant volume of hazardous solvents, including acetonitrile, methanol, and methylene chloride, is utilized as extracting agents and in liquid chromatography mobile phases, posing environmental risks [147].

Recently, high-resolution mass spectrometers (HRMS), including Orbitrap and time-of-flight (TOF) analyzer systems, have enhanced the application of mass spectrometry in analytical methods due to their exceptional capacity to detect a theoretically infinite number of compounds in full-scan mode, along with their structural information [79]. LC-HRMS provides the ability to simultaneously monitor an infinite array of compounds [148]. This approach is highly beneficial for the concurrent measurement of unidentified substances, including metabolites and transformation products in environmental samples, and it is utilized for untargeted analysis and suspect screening [79]. Moreover, LC-HRMS is an effective instrument for identifying unidentified compounds in complex matrices. HRMS is optimal for non-targeted food safety testing owing to its superior mass accuracy, resolution, scan speed, and sensitivity in full-scan mode. This has resulted in the creation of rapid and extensive multi-residue screening techniques, such as LC/Q-TOF-HRMS screening, for more than 600 multi-class compounds. In regulatory contexts, HRMS screening is facilitated by identification criteria and the Screening Detection Limit concept, establishing LC–HRMS as a versatile tool for both targeted quantification and exploratory contaminant detection in complex food matrices [149,150]. Omics-based methodologies, such as metabolomics and proteomics, provide robust tools for the untargeted assessment of pesticide effects and residue patterns, facilitating a comprehensive knowledge of exposure routes and ecotoxicological consequences [151].

Supercritical fluid chromatography (SFC) substitutes the majority of liquid mobile phases with high-density compressed gas for the separation of intricate mixtures. Carbon dioxide (CO_2_) is predominantly employed as the mobile phase in SFC because of its advantageous characteristics, including a low critical point, little toxicity, and low flammability. CO_2_ does not harm hardware and may be used with many liquid organic solvents [48]. In comparison to HPLC, UPLC, and GC, it diminishes the volume of organic solvents and analysis duration, aligning with the principles of environmental conservation and green chemistry [152]. The SFC-MS/MS method is commonly employed for the separation of non-volatile or thermally unstable pesticides and for quantifying chiral or achiral chemical compounds in biological samples, owing to its advantages in speed, sensitivity, and cost-effectiveness [17].

The comprehensive monitoring of pesticide exposure requires the detection of both parent chemicals and their metabolites, employing sensitive analytical methods capable of identifying both at minimal levels. Upon release into the environment, a pesticide can go through metabolic processes, resulting in the simultaneous existence of the parent molecule and its metabolites, which may have toxicological significance. European and national reference laboratories should employ suspicious screening methodologies to assess the effects of pesticide metabolites. Furthermore, authorities might provide access to metabolite standards to facilitate the accurate identification and quantification of important pesticide metabolites [153]. Global concerns over environmental contamination by fluorinated compounds, particularly per- and polyfluoroalkyl substances (PFAS), have arisen due to their established detrimental effects on human health, wildlife, and ecosystem integrity [154]. Although methods for PFAS detection are available, the analysis is challenging, requiring an intensive procedure and expensive equipment. Various analytical methods are effective tools for separation and fractionation in PFAS analysis, including GC, LC, and HPLC. GC or LC is frequently coupled with MS, or tandem mass spectrometry (LC-MS/MS, GC-MS), for the detection of both particular and non-specific per- and PFAS [155].

The primary factors that researchers analyze when evaluating the validity of a technique are the limits of detection (LOD) and limits of quantification (LOQ). LODs represent the minimum concentration of a pesticide that can be identified with acceptable precision and accuracy under defined testing circumstances. Table 3 shows current research in which various chromatographic techniques were effectively employed, together with the LODs and LOQs for the examined pesticide residues.

In mass spectrometry, quantification can be accomplished with external or internal standards. An external standard depends on a calibration curve established using a pure analyte standard assessed under equivalent experimental circumstances. The advantages include accessibility and the lack of overlap with endogenous species, as the standard is examined independently from the sample. Nonetheless, it is susceptible to variability arising from sample preparation, extraction recovery, and matrix effects, which might modify ionization responses in comparison to the calibration solution. In contrast, internal standardization entails the direct incorporation of a structurally analogous or isotopically tagged substance into the sample, facilitating the adjustment for extraction losses and ionization inconsistencies. This method provides enhanced correction for matrix-dependent variations and increases quantitative accuracy across various sample types when the internal standard closely resembles the analyte’s activity [177,178]. In summary, external standardization delivers superior curve precision but is susceptible to matrix-induced variability, whereas internal standardization enables effective correction for these effects but is limited by the requirement for meticulously chosen, reliable, and frequently expensive standards. Consequently, the selection signifies a compromise between accuracy and feasibility, contingent upon the analyte–matrix combination.

Several efforts have been undertaken to mitigate matrix effects. For example, the precision of quantitative analysis may be enhanced by the utilization of internal standards in multi-residue analysis employing QuEChERS and LC-MS/MS for food products [81].

### 4.2. Rapid Technologies

#### 4.2.1. Biosensors

Screening techniques, such as biosensors, must accomplish high throughput in tested samples and a brief analysis period while ensuring adequate detectability with limits of detection lower than MRLs. Nonetheless, pesticide residues are often removed utilizing organic solvents and extensive sample preparation procedures. This presents a significant difficulty for screening technologies, as they often involve selective biomolecules that exhibit specific tolerance to organic solvents [26].

Biosensors are analytical instruments that integrate biological sensing elements with physical and chemical transducers, enabling quantitative or semi-quantitative studies. A biosensor consists of a receptor, a transducer, and a biorecognition element that identifies specific target molecules in a medium [179]. Various biosensors possess distinct physical and chemical characteristics, which can exert particular effects on the target to facilitate identification and signal generation for analysis [180]. They provide a straightforward, economical, and dependable method for detecting pesticides in food matrices to guarantee consumer food safety.

Transducers have a key role in identifying a certain target material for biosensors. The primary role is to transform an analytical signal into an informative reading signal. Consequently, several research domains have assessed various methodologies. The most used transducers are optical (colorimetric and fluorescence), electrochemical, amperometric, and SERS (surface-enhanced Raman spectroscopy). Selecting the appropriate transducer for the biosensor is crucial, particularly in the presence of nanomaterials, since it directly affects the detection sensitivity of the target analyte [179]. For example, OPPs are detected using a variety of techniques, including chemiluminescence, electrochemical, colorimetric, and fluorescence [181]. Table 4 provides several biosensors utilized for the detection of pesticides in food products. Research on multi-residue pesticide biosensors that can simultaneously detect several pesticides is worth mentioning. Zhao et al. created an electrochemical biosensor utilizing nanogold/mercaptomethamidophos to identify 12 organophosphate pesticides in cabbage and apple samples [182].

Establishing specific research and development strategies is essential for the advancement of multi-residue sensors. The selection of detection technology must be precise; electrochemical sensors detect variations in current or potential linked to the binding of target substances, optical sensors utilize fluorescence or absorption characteristics to identify molecules, and biorecognition-based platforms, including enzymes, antibodies, or aptamers, facilitate the selective recognition of distinct residues. Additionally, the functionalization of sensors by the incorporation of specialized receptors or their integration into a multi-analytical chip enables the concurrent detection of several chemicals. Integration with microfluidics facilitates miniaturization and rapid sample analysis, enabling the manipulation of minuscule sample volumes. The utilization of genetically engineered enzymes, highly specific antibodies, microbial cells, aptamers, and molecularly imprinted polymers for biosensor design can significantly enhance sensor selectivity. Moreover, the advancement of “lab-on-a-chip” systems that integrate electrochemistry, optics, and biorecognition enables the swift and concurrent detection of many signals. The validation of sensors by testing on actual samples and comparison with conventional laboratory procedures guarantees the accuracy, sensitivity, and resilience of the system [26,183,184]. Recent research has led to portable devices for rapid pesticide detection in the field, addressing the constraints of laboratory methods. Advancements in technology have enabled smartphones and wearable gadgets with high-resolution cameras and robust image processing capabilities. These devices may now be utilized as conveniently portable and accessible tools for colorimetric analysis. The integration of colorimetric detection with smartphones and wearables has several benefits. Firstly, it obviates the necessity for cumbersome and costly laboratory apparatus, rendering it more economical and accessible, especially in resource-constrained environments or for immediate testing. Furthermore, it facilitates real-time analysis and remote monitoring, enabling rapid reaction and data dissemination [185]. Chen et al. presented a smartphone-based colorimetric sensor for on-site pesticide analysis in agricultural settings, whereas Li et al. described a portable paper-based device combined with a smartphone camera that enabled quick detection at the point of application [186,187].

Smartphone assays may provide preliminary on-site screening, eliminating the need for sample collection and transportation, while promptly producing a sample ID and delivering screening results with a specified false positive/false negative rate [26].

**Table 4:** Transductor-based biosensors used for pesticide detection in the food matrix.

Transductor	Food Matrix	Pesticide	Characteristics	LOD	Ref.
Electrochemical	-	Paraoxon	20 consecutive measurements → the operational stability = 94.13% Storage stability (60 days) = 70%	0.17 nM	[188]
Electrochemical	Cabbage	Dichlorvos	Good stability	0.23 nM	[189]
Electrochemiluminescence	Rape Pineapple	Methyl parathion	Highly sensitive	-	[190]
Fluorescence	Tea	Quinalphos Thiamethoxam Propargite Hexaconazole	High stability Simultaneous detection of 4 pesticides in a single sample	0.2 ng/mL	[140]
Fluorescence	Orange juice Apple juice	Ethyl parathion	High sensitivity Simpler synthetic protocol	2.40 pM	[45]
Colorimetric	Fruits Vegetables	Chlorpyrifos Profenofos Cypermethrin	High sensitivity Good linear response	0.235 mg/L 4.891 mg/L 4.053 mg/L	[191]
Colorimetric	Pear Rice Cabbage	Parathion	Wide linear range 0.01–50 µg·L^−1^	2.04 ng·L^−1^	[192]
SERS	-	Profenofos Acetamiprid Carbendazim	Stable and significantly short measurement time Low detection limit	0.0021 ng mL^−1^ 0.0046 ng mL^−1^ 0.0061 ng mL^−1^	[193]
SERS	Apples	Methyl parathion	Paper-based substrate Superior reproducibility Good stability and sensitivity	0.011 µg/cm^2^	[44]

SERS, surface-enhanced Raman scattering.

Nanomaterials are often employed in biosensors due to their substantial enhancement in performance, facilitating quicker, more efficient, and cost-effective detection. The enhancement in performance arises from their distinctive optical and electrical characteristics, which yield a high contact surface-to-volume ratio, elevated electrical conductivity, catalytic activity, biocompatibility, and ease of modification with functional groups, including gold nanoparticles (AuNPs), silver nanoparticles (AgNPs), silver nanowires (AgNWs), gold nanorods (AuNRs), gold nanostars (AuNSs), carbon nanotubes (CNTs), copper nanowires (CuNWs), and multi-wall carbon nanotubes (MWCNTs). In addition to pure materials, several hybrid nanostructures have also been examined [179]. Arsawiset et al. developed a paper-based analytical device utilizing CuO nanoparticle nanozymes for the quick detection of malathion in fruits and vegetables, attaining a linear detection range of 0.1–5 mg/L, a detection limit of 0.08 mg/L, and an analysis duration of roughly 10 min [194]. Qin et al. proposed an electrochemical biosensor utilizing a perovskite/AuNPs composite, which markedly improved electron transport and conductivity, enabling ultra-sensitive detection of fenitrothion with a detection limit of 0.034 µg/L in food products [195].

Electrochemical biosensors draw researchers interest due to their high sensitivity, excellent stability, ease of downsizing, low cost, and rapid detection; biosensors based on AChE are particularly promising. Nanomaterials are optimal for the fabrication of these AChE sensors [189]. The basic function of enzyme-based electrochemical biosensors is to detect and analyze changes in electrical signals. This signal is the outcome of an enzymatic reaction that produces or reduces an electroactive species [188]. Because of their high sensitivity and user-friendliness, electrochemical transducers were the most often used [179].

Fluorescence offers benefits over absorbance-based techniques, including sensitivity, selectivity, and a shorter detection time. Because of interactions between fluorophores and surface plasmons in metallic nanostructures, its sensitivity can be up to 100 times higher than that of absorbance methods. Its high selectivity is caused by the fluorescent molecule, which often has many emission spectra [196]. Fluorescence-based optical signaling of organophosphates, compared to alternative approaches, has proven to be more advantageous due to its relative simplicity, overall rapidity, and ultra-sensitivity [45].

Colorimetric transducers are more affordable, portable and lighter, have a smaller sample size, and need less equipment compared to conventional techniques [179]. Gold nanoparticles (AuNPs) have been extensively utilized as effective signal transducer elements in the creation of colorimetric pesticide sensors [5]. Regrettably, most colorimetric analytical platforms employ conventional sample preparation processes, underscoring the necessity to automate and streamline sample pretreatment to enhance the application of these approaches in practical settings [26]. Researchers utilizing this type of transducer have noted challenges, including printing reproducibility, image capture, difficulty in differentiating separate components of a combination, and stability issues [179].

SERS employs precious metal nanoparticles and the principle of electromagnetic field enhancement to induce Raman enhancement in molecules adsorbed on the surface [193]. Currently, it has been utilized in several areas, including food safety, life sciences, environmental monitoring, and the chemical industry, and it may be applicable in the quick detection of pesticide residues [180]. The uniformity and sensitivity of the signal are critical variables for assessing the SERS technique, which is associated with SERS substrates [44].

Currently, the majority of quantitative analyses of pesticide residues on the surfaces of fruits and vegetables utilizing SERS detection technology rely on the linear quantification of a singular, distinctive peak of pesticides. Nonetheless, throughout the detection process, the Raman characteristic peak of pesticide contaminants is susceptible to small shifts caused by nonlinear variables, including instrument and ambient noise [180]. Despite its promise for direct detection of pesticides at low levels in liquid samples or on solid food surfaces, the use of SERS for identifying internalized pesticides in complex solid food matrices remains challenging [141]. The integration of Raman spectroscopy with other detection methods can enhance detection capabilities. Nie et al. utilized the aptamer pesticide structure as the target for Raman spectroscopy, enabling the specific detection of malathion. Alami et al. integrated Raman spectroscopy with an enzyme inhibition technique. Consequently, the advancement of Raman signal enhancement substrates that are appropriate for detecting a broader range of pesticide residues at a reduced cost and with greater stability is essential for facilitating the application of Raman spectroscopy in pesticide residue detection [180,197,198]. SERS is a very promising method for the direct detection of pesticides at trace levels in liquid samples or on solid surfaces after straightforward extraction to enhance analyte concentration [199].

#### 4.2.2. ELISA (Enzyme-Linked Immunosorbent Assays)

Immunoassays are generally recognized as an efficient technique for the quick detection of pesticide residues, utilizing antibodies as the recognition element. They are primarily categorized into colorimetry, optics, and electrochemistry tests based on the signal transductions. Colorimetric immunoassays have been widely favored due to their high specificity, cost-effectiveness, operational simplicity, and rapid reaction time. ELISA is considered the gold standard among colorimetric immunoassays due to its automation, high throughput, and scalability [200].

Detection methods utilizing ELISA may employ a double antibody sandwich technique or a capture approach—both of which are noncompetitive—or a competitive method. In this technique, the antibody and the target analyte generate a complex, and the detection signal is directly proportional to the concentration of the target analyte, often suitable for the detection of macromolecular antigens. Pesticides, being a category of small molecular compounds with singular antigenic characteristics, can exclusively bind to one antibody; hence, the competitive detection approach is typically employed. The detection signal exhibits a negative correlation with the concentration of the target analyte [180].

Abraxis Life Technologies™ (Los Angeles, CA, USA) delivers field and laboratory ELISA testing kits for many pesticides analyzed in diverse matrices specified in the National Environmental Methods Index. Multiple studies have demonstrated the efficacy of Abraxis pesticide kits, particularly for glyphosate analysis [201]. López Dávila et al. demonstrated that ELISA is an analytical method for the rapid identification, control, and monitoring of pesticide residues in tomatoes, sweet peppers, and cucumbers prior to chromatographic analysis (GC or LC) [202]. In another research study, the developed ELISA demonstrated an accuracy of 114% recovery with a 3% coefficient of variation for OPPs/Carbamates and 115% recovery with a 4.0% coefficient of variation for pyrethroids [201].

Researchers created very sensitive and straightforward immunoassays for detecting small quantities of imidacloprid by using phage-borne peptides as alternatives to chemically generated antigens. Two peptides were extracted from phage display libraries, competing with imidacloprid for affinity to the monoclonal antibody 3D11. Optimizations were performed on a phage–enzyme-linked immunosorbent assay (P-ELISA) and two phage time-resolved fluoroimmunoassays (P-TRFIAs), resulting in IC50 values of 0.067, 0.085, and 0.056 ng/mL, respectively, demonstrating almost four times increased sensitivity compared to prior methodologies [203].

A colorimetric ELISA test kit was employed to directly identify organophosphates and carbamates, whereas the analysis of pyrethroids was conducted using paramagnetic particles conjugated to antibodies particularly designed for pyrethroid detection. The samples were also tested by chromatography to validate the favorable results. The ELISA kits facilitated the detection of organophosphate, carbamate, and pyrethroid residues in the collected samples. The evaluated ELISA kits demonstrated quantification capabilities at levels below the detection threshold of the employed chromatographic methods. Linear correlations between the quantified values derived from the chromatographic approach and the findings obtained from the pyrethroid ELISA test kits were noted [202].

Alongside ELISA techniques, lateral flow immunoassay (LFIA) is another traditional immunochemical approach that has been extensively utilized for the on-site detection of residues. LFIA has several benefits, including user-friendliness, little time investment, simplicity, affordability, and the capability for high-throughput screening of multiple target analytes. The predominant LFIA technique utilizes gold nanoparticles (AuNPs) as reporters for colorimetric detection. A lateral flow immunoassay (LFIA) employing a widely specific anti-adamantane monoclonal antibody has been established for five adamantanes, with optical LODs ranging from 0.1 to 10 µg/kg, comparable to ELISA findings. To enhance the sensitivity of LFIA, other labels, such as fluorescent nanoparticles, have been utilized in its synthesis. An ultrasensitive fluorescent lateral flow immunoassay has been developed using a nonspecific monoclonal antibody [204].

## 5. Conclusions

Several studies have been conducted in recent years to evaluate the pesticide residue levels in food products. The advancement of analytical techniques has resulted in enhanced sensitivity and specificity in detection, facilitated by microextraction technologies that surpass traditional extraction approaches. Simultaneously, chromatographic techniques like HPLC and GC, when combined with high-performance detectors, provide accurate and quantitative detection of pesticide residues. Accurate detection of pesticide levels in various food types requires adequate extraction, clean-up, and enrichment of samples.

Additionally, the advancement of biosensors suggests a potential avenue, providing rapid and effective options for detecting pollutants in food items. They have the potential to revolutionize several food safety applications, enhancing regulatory compliance and safeguarding public health with increased efficacy. Intelligent biosensors will serve as a formidable instrument in enhancing risk management and guaranteeing optimal standards of food safety and quality in the present and future. Despite their advantages, biosensors encounter difficulties, including environmental interferences, signal reproducibility challenges, false positives, stability concerns, elevated material prices, and protracted baseline needs. Nonetheless, they are generally far less expensive and more portable than traditional chromatographic–mass spectrometric techniques, rendering them appealing for rapid screening.

Unfortunately, the limited availability of essential components (antibodies/enzymes for immunoassays, specialized sorbents or SPME fibers, biosensor recognition elements/nanomaterials) may restrict the replication and broad implementation of investigations among the scientific community. We support the fair dissemination of technological advances through open licensing for university research and partnerships with nonprofit laboratories to promote scientific advancement and widespread access to established methodologies.

The opportunities for enhancing food safety by reducing pesticide residues encompass the ongoing reassessment of pesticides, higher use of safer and less toxic alternatives, extensive education and training for producers, international cooperation, and the development of effective pesticide elimination strategies.

From a One Health perspective, current analytical advancements provide integrated surveillance across the food–environment–biomonitoring continuum and enhance cumulative/mixture exposure evaluation essential to biodiversity and antimicrobial resistance. Aligning these metrics with regulatory frameworks provides decisions that are legally acceptable and intervention-oriented, while accounting for socioeconomic drivers promotes effective risk-reduction approaches.

## Figures and Tables

**Table 1:** Advantages and limitations of sample preparation techniques in pesticide residue analysis.

Extraction	Advantages	Limitations
MAE	High extraction efficiency Green and eco-friendly Automation Low solvent consumption	Poor extraction performance for non-polar/volatile compounds Not suitable for thermally unstable analytes
ASE	Reduced solvent and time consumption Simple operation and eco-friendly	High cost of equipment and maintenance Handling of extraction cells can be challenging
SPE	Simple, cost-effective, widely available Variety of sorbents for diverse pesticide properties	Risk of co-eluting interferences
dSPE	Requires minimal equipment Efficient cleanup of fatty acids, pigments, and sugars	Risk of analyte loss during cleanup
MSPD	Low sample and solvent consumption Suitable for multi-residue analysis	Requires optimization Sorbent handling
SPME	Solvent-free/minimal solvent Simple and flexible Reusable fibers	Limited commercial availability of specialized fibers
SFE	Reduced solvent use Prevents oxidation/degradation Environmentally friendly	Mainly extracts non-polar compounds Requires high-purity CO_2_ Expensive equipment
QuEChERS	Fast and simple Minimal equipment Broad analyte range	May need modifications for fatty/complex matrices
HF-LPME	Very low solvent usage Eco-friendly	Limited mainly to polar analytes
DLLME	Suitable for hydrophobic analytes Fast Low cost	Restricted solvent choices Involves multiple stages
SDME	Very eco-friendly (few µL solvents) Simple and cost-effective	Reduced reproducibility
CSDF-ME	Minimal solvent use	Limited validation

**Table 2:** Sample pretreatment for detecting pesticide residues in various food matrices (2020–2025).

Pretreatment	Food Matrix	Pesticide	EF	ER	RR (%)	Ref.
MAE	Maize	Atrazine Glyphosate Mesotrione	-	-	80–98	[71]
MAE	Fruits and vegetables	Mancozeb	-	-	81–112	[73]
MAE	Apples	Thiamethoxam	-	-	61–112	[70]
ASE	Soy products	230 pesticides	-	-	70–120	[74]
ASE	Corn flour	Glyphosate	-	-	109.19 ± 8.26	[114]
SPE	Fruits and vegetables	Fluindapyr + metabolites	-	-	71–118	[115]
SPE	Packed fruit juice	Ametryn Chlorpyrifos Clodinafob-propargyl Fenpropathrin Oxadiazon Diniconazole Penconazole	452–751	45–75	85–101	[116]
dSPE	Orange juice	OPPs	-	-	95.35–110.75	[117]
dSPE-DLME	Strawberries	Hexaconazole Oxadiazon Tebuconazole Clodinafop-propargyl Difenoconazole	365–405	73–81	-	[118]
MSPD	Eggplant Capsicum Apple gourd Cauliflower Sponge gourd	Diafenthiuron Lufenuron Azoxystrobin Difenoconazole Chlorothalonil	-	-	88.5–116.9	[119]
MSPD	Corn	Triazines	-	-	92.6–104.7	[120]
SPME	Tea	OPPs	-	-	73.12–101.20	[121]
SPME	Fruits and vegetables	OPPs	-	-	82.6–118	[122]
HS-SPME	Grapefruit Cucumber	OPPs	-	-	85–118	[123]
SFE	Brown rice	OPPs Pyrethroid Dithiolane	-	-	96.4–105.0	[124]
SFE	Green onion	Acetamiprid Clothianidin Dinotefuran Imidacloprid Thiacloprid Thiamethoxam	-	-	70–120	[125]
QuEChERS	Mandarin Potato Green Pepper Hulled rice Soybean	Triflumezopyrim	-	-	89.7–104.3	[126]
QuEChERS	Vegetables	*β*-HCH, Υ-HCH Cypermethrin Profenophos 4-Nonylphenol p,p′-DDD, p,p′-DDTs	-	-	69–114	[127]
QuEChERS	Muscle chicken breast fillets	α-endosulfan Cypermethrin Endosulfan sulfate Permethrin DDT	-	-	71.2–118.80	[99]
DLLME + dSPE	Tomato Lettuce Carrot Celery	Hexaconazole Chlorpyrifos Diazinon Tebuconazole Diniconazole	380–430	79–86	90–103	[128]
DLLME + QuEChERS	Yogurt	OCPs OPPs Dinitroanilines Carbamates and pyrethroids Triazines Chloracetamides Dicarboximides Azoles	5–16	-	70–120	[129]
HF-LPME	Canned drinks	OPPs	-	-	73.6–94.8	[102]
HF-PLM	Rice	OCPs	67.4–73.5%	76.277–81.499	86.0–92.9	[130]
CSDF-ME + UAE	Apple Strawberry Cucumber Tomato	OPPs	21–205%	-	83.0–108.0	[131]
CSDF-ME	Juice	OPPs	102–380 µg L^−1^	17–51	83–105	[110]
CSDF-ME	Grape juice	OPPs	510–960	25.5–48.0%	90–110	[112]

UAE, ultrasound-assisted extraction; HF-PLM, hollow fiber-protected liquid-phase microextraction; HS-SPME, headspace solid-phase microextraction.

**Table 3:** Chromatography techniques used to detect pesticide residues in food products (2020–2025).

Detector	Food Matrix	Pesticide	LOD	LOQ	Ref.
1. LC-MS/MS 2. GC-MS/MS	Honey	Carbendazim Thiabendazole Azoxystrobin Chlorpyrifos Imidacloprid	10.0001–0.0004 mg/kg 0.001–0.004 mg/kg	0.0002–0.0008 mg/kg 0.002–0.008 mg/kg	[156]
LC-MS/MS	Vine leaves	512 pesticides	-	-	[142]
LC-MS/MS	Mandarins	440 pesticides	<0.01 mg kg^−1^	-	[143]
LC-MS/MS	Pistachio	112 pesticides	0.003 mg/kg	0.01 mg/kg	[157]
LC-Q-TOF/MS	Mango	345 pesticides	-	0.5 to 20 µg/kg	[158]
HPLC	Fruit juice and white wine	Carbofuran Carbaryl Isoprocarb Diethofencarb	0.3 µg/L	-	[159]
GC	Soybean	No residues of the target pesticides were detected	-	-	[107]
GC	Fruits and vegetables	Mancozeb	0.003 kg^−1^	0.01 mg kg^−1^	[73]
GC-MS	Oat flour	Triadimenol Flutriafol λ-cyhalothrin Difenoconazole Azoxystrobin	1.7–12.9 µg kg^−1^	5.73–43.0 µg kg^−1^	[145]
GC-MS	Spinach	108 pesticides	0.005–0.01 µg/g	0.01–0.025 µg/g	[160]
GC-MS	Rice	15 pesticides	0.10–1.46 µg kg^−1^	0.390–4.85 µg kg^−1^	[161]
GC-FID	Tomato	Spiromesifen	0.0015 µg mL^−1^	0.006 µg mL^−1^	[162]
GC-ECD	Milk	OCPs	3.7 to 4.8 µg L^−1^	12–16 µg L^−1^	[163]
GC-ECD	Melon Cucumber	Pyraclostrobin Difenoconazole Dimethomorph Azoxystrobin	-	0.01–0.05 mg/L	[164]
GC-FPD	Beet	35 pesticides	0.0047–0.0261 mg/kg	0.0143–0.0790 mg/kg	[146]
GC-MS/MS LC-MS/MS	Vegetables	80 pesticides	0.0004–0.0023 mg kg^−1^	0.0008–0.0047 mg kg^−1^	[165]
GC-MS/MS	Lime Lemon	45 pesticides	1.56–25.23 ng/mL	4.72–76.47 ng/mL	[166]
GC-MS/MS	Seafood	44 pesticides	2–3 ng/g	7–10 ng/g	[167]
GC-MS/MS	Rice	Triazophos Dichlorvos Chlorpyrifos Malathion	3.4–5.4 µg/kg	20 µg/kg	[168]
HPLC-MS/MS	Rice	Acetamiprid Parathion Profenofos Bixafen	10 µg/kg	-	[169]
HPLC-FL/UV GC-MS/ECD/NPD	Tomatoes	180 pesticides	5–10 µg/kg	10–20 µg/kg	[170]
HPLC-HRMS	Grapes	92 pesticides	4.88–120.16 µg L^−1^	14.86–308.01 µg L^−1^	[171]
UHPLC-MS/MS GC-MS/MS	Kumquat fruits	16 insecticides 7 fungicides 5 acaricides 2 plant growth modulators	-	-	[172]
UHPLC-MS/MS	Fruits and vegetables	Fluindapyr + 5 metabolites	0.0001–0.0002 mg/L	0.0003–0.0006 mg/L	[115]
UHPLC-MS/MS	Apples	Pyraclostrobin	-	0.01 mg kg^−1^	[173]
UHPLC-MS/MS	Dates	Carbamates	0.01–0.005 µg kg^−1^	0.003–0.04 µg kg^−1^	[174]
SFC-MS/MS	Rice Wheat Maize	9 pesticides	0.01–42.9 µg/kg	0.4–101.8 µg/kg	[48]
SFC-MS/MS	Jujube Peach Grape Pear	Metconazole	4.30–95.9 ng/kg	10.5–143.2 ng/kg	[175]
SFC-IM-Q-TOF/MS	Yam Potato	20 pesticides	0.1–8.8 ng/mL	0.8–29.4 ng/mL	[152]
LC-HRMS/MS	Cider	18 pesticides	-	0.2 µg L^−1^	[176]
LC-HRMS	Cereals and Grains	730 pesticides	-	5–20 µg/kg	[148]
1. GC-MS 2. HPLC-UV	Cereal (wheat, rice, corn)	323 pesticides	1. 0.0025–0.005 mg kg^−1^ 2. 0.003–0.027 mg kg^−1^	-	[31]

Q-TOF, quadrupole-time-of-flight mass spectrometry; FL, fluorescence; UV, ultraviolet; UHPLC, ultrahigh-performance liquid chromatography; IM-Q-TOF/MS, ion mobility quadrupole time-of-flight mass spectrometry.

## Data Availability

No new data were created or analyzed in this study. Data sharing is not applicable.

## Abbreviations

The following abbreviations are used in this manuscript:

MRLs	Maximum Residue Limits
GC	Gas Chromatography
MS	Mass Spectrometry
LC	Liquid Chromatography
HPLC	High-Performance Liquid Chromatography
OCPs	Organochlorine Pesticides
OPPs	Organophosphates Pesticides
DDT	p,p′-dichlorodiphenyltrichloroethane
DDE	Dichlorodiphenyldichloroethylene
2,4-D	2,4-dichlorophenoxyacetic acid
EPA	Environmental Protection Agency
IARC	International Agency for Research on Cancer
AChE	Acetylcholinesterase
EFSA	European Food Safety Authority
GAP	Good Agricultural Practices
HBGVs	Health-Based Guidance Values
ADI	Acceptable Daily Intake
UL	Tolerable Upper Intake Level
SFC	Supercritical Fluid Chromatography
MAE	Microwave-Assisted Extraction
PFAS	Polyfluoroalkyl Substances
LOD	Limit Of Detection
LOQ	Limit Of Quantification
ASE	Accelerated Solvent Extraction
QuEChERS	Quick, Easy, Cheap, Rugged, Effective and Safe
PSA	Primary Secondary Amine
SPE	Solid Phase Extraction
dSPE	Dispersive Solid Phase Extraction
UHPLC	Ultra-High-Performance Liquid Chromatography
SPME	Solid-Phase Microextraction
MSPD	Matrix Slid-Phase Dispersion
BiT-MSPD	Balls-in-Tube Matrix Solid-Phase Dispersion
SFE	Supercritical Fluid Extraction
HF-LPME	Hollow Fiber Liquid-Phase Microextraction
PF	Pre-concentration Factor
EF	Enrichment Factor
ER	Extraction Recovery
RR	Recovery Rate
DLLME	Dispersive Liquid–Liquid Microextraction
SDME	Single Drop Microextraction
CSDF-ME	Continuous Sample Drop Flow Microextraction
CFME	Continuous Flow Microextraction
LPME	Liquid-Phase Microextraction
UAE	Ultrasound-Assisted Extraction
HF-PLM	Hollow Fiber-Protected Liquid-Phase Microextraction
G-HF-LPME	Graphene-Reinforced Hollow Fiber Liquid-Phase Microextraction
G-HC-LPME	Gas-assisted Hollow Fiber Liquid-Phase Microextraction
HS-SPME	Headspace Solid-Phase Microextraction
NPD	Nitrogen Phosphorus Detector
ECD	Electron Capture Detector
FID	Flame Ionization Detector
FPD	Flame Photometric Detector
HRMS	High-Resolution Mass Spectrometer
Q-TOF/MS	Quadrupole-Time-of-Flight Mass Spectrometry
FL	Fluorescence
SERS	Surface-Enhanced Raman Spectroscopy or Scattering
UV	Ultraviolet
ELISA	Enzyme-Linked Immune Sorbent Assays

## References

[1] Ouyang M., Liu T., Yuan X., Xie C., Luo K., Zhou L. (2025). Nanomaterials-Based Aptasensors for Rapid Detection and Early Warning of Key Food Contaminants: A Review. Food Chem..

[2] Scutarașu E.C., Trincă L.C. (2023). Heavy Metals in Foods and Beverages: Global Situation, Health Risks and Reduction Methods. Foods.

[3] Popa I.D., Şchiriac E.C., Ungureanu D., Cuciureanu R. (2012). Immune Response in Rats Following Administration of Honey with Sulfonamides Residues. Rev. Rom. Med. Lab..

[4] Hashimi M.H., Hashimi R., Ryan Q. (2020). Toxic Effects of Pesticides on Humans, Plants, Animals, Pollinators and Beneficial Organisms. Asian Plant Res. J..

[5] Bashir K., Shikha S., Rattu G., Jan K., Krishna P.M., Pattanayek S.K. (2024). Pesticide Residues and Their Detection Techniques in Foods Using Sensors—A Review. J. Food Sci. Technol..

[6] Singhal M., Jadhav S., Sonone S., Singh Sankhla M., Kumar R. (2021). Microalgae Based Sustainable Bioremediation of Water Contaminated by Pesticides. Biointerface Res. Appl. Chem..

[7] Hoffmann V., Paul B., Falade T., Moodley A., Ramankutty N., Olawoye J., Djouaka R., Lekei E., de Haan N., Ballantyne P. (2022). A One Health Approach to Plant Health. CABI Agric. Biosci..

[8] Botnaru A.A., Lupu A., Morariu P.C., Pop O.L., Nedelcu A.H., Morariu B.A., Cioancă O., Di Gioia M.L., Lupu V.V., Avasilcai L. (2025). Balancing Health and Sustainability: Assessing the Benefits of Plant-Based Diets and the Risk of Pesticide Residues. Nutrients.

[9] Dhuldhaj U.P., Singh R., Singh V.K. (2023). Pesticide Contamination in Agro-Ecosystems: Toxicity, Impacts, and Bio-Based Management Strategies. Environ. Sci. Pollut. Res..

[10] Falkenberg T., Ekesi S., Borgemeister C. (2022). Integrated Pest Management (IPM) and One Health—A Call for Action to Integrate. Curr. Opin. Insect. Sci..

[11] Gavahian M., Pallares N., Al Khawli F., Ferrer E., Barba F.J. (2020). Recent Advances in the Application of Innovative Food Processing Technologies for Mycotoxins and Pesticide Reduction in Foods. Trends Food Sci. Technol..

[12] Garud A., Pawar S., Patil M.S., Kale S.R., Patil S. (2024). A Scientific Review of Pesticides: Classification, Toxicity, Health Effects, Sustainability, and Environmental Impact. Cureus.

[13] Louppis A.P., Kontominas M.G. (2024). Recent Developments (2020–23) on the Use of LC in the Determination of Food Contaminants. Separations.

[14] Botnaru A.A., Lupu A., Morariu P.C., Jităreanu A., Nedelcu A.H., Morariu B.A., Anton E., Di Gioia M.L., Lupu V.V., Dragostin O.M. (2025). Neurotoxic Effects of Pesticides: Implications for Neurodegenerative and Neurobehavioral Disorders. J. Xenobiot..

[15] Caba I.-C., Ștreangă V., Dobrin M.-E., Jităreanu C., Jităreanu A., Profire B., Apotrosoaei M., Focșa A.-V., Caba B., Agoroaei L. (2022). Clinical Assessment of Acute Organophosphorus Pesticide Poisoning in Pediatric Patients Admitted to the Toxicology Emergency Department. Toxics.

[16] Boneva I., Yaneva S., Danalev D. (2021). Development and Validation of Method for Determination of Organophosphorus Pesticides Traces in Liver Sample by GC-MS/MS-Ion Trap. Acta Chromatogr..

[17] Tucker S., Dumitriu G.-D., Teodosiu C. (2022). Pesticides Identification and Sustainable Viticulture Practices to Reduce Their Use: An Overview. Molecules.

[18] Sel S., Er E.Ö., Koyuncu İ. (2024). Development of an Analytical Method for the Determination of Pesticides in Tropical Fruits by LC-QTOF-MS/MS after QuEChERS Extraction Sample Cleanup and DLLME Preconcentration. Methods Appl. Fluoresc..

[19] Chormey D.S., Zaman B.T., Kasa N.A., Bakırdere S. (2020). Liquid Phase Microextraction Strategies and Their Application in the Determination of Endocrine Disruptive Compounds in Food Samples. TrAC Trends Anal. Chem..

[20] Rani P., Nanda B.P., Narang R.K., Bhatia R. (2024). Advancements in Solvent Microextraction: Recent Developments and Diverse Applications in the Modern Era. Sep. Sci. Plus.

[21] Rodríguez-Ramos R., Lehotay S.J., Michlig N., Socas-Rodríguez B., Rodríguez-Delgado M.Á. (2020). Critical Review and Re-Assessment of Analyte Protectants in Gas Chromatography. J. Chromatogr. A.

[22] Brinco J., Guedes P., Gomes da Silva M., Mateus E.P., Ribeiro A.B. (2023). Analysis of Pesticide Residues in Soil: A Review and Comparison of Methodologies. Microchem. J..

[23] Shalaby A.A., El-Sheikh E.S.A., Refaat A.M., Ragheb D.A. (2022). Residue Analysis and Associated Risk Assessment of Hexythiazox and Spinosad Applied on Strawberry Plants. Egypt. J. Chem..

[24] Gai T., Nie J., Ding Z., Wu W., Liu X. (2023). Progress of Rapid Detection of Pesticides in Fruits and Vegetables. Front. Food Sci. Technol..

[25] Moschopoulou G., Tsekouras V., Mercader J.V., Abad-Fuentes A., Kintzios S. (2024). Development of a Portable Cell-Based Biosensor for the Ultra-Rapid Screening for Boscalid Residues in Lettuce. Biosensors.

[26] Tsagkaris A.S., Pulkrabova J., Hajslova J. (2021). Optical Screening Methods for Pesticide Residue Detection in Food Matrices: Advances and Emerging Analytical Trends. Foods.

[27] Ansari I., Magdy El-Kady M., Muniyan S. (2021). A Review on the Fatal Impact of Pesticide Toxicity on Environment and Human Health 16. Global Climate Change.

[28] Dawson A.H., Eddleston M., Senarathna L., Mohamed F., Gawarammana I., Bowe S.J., Manuweera G., Buckley N.A. (2010). Acute Human Lethal Toxicity of Agricultural Pesticides: A Prospective Cohort Study. PLoS Med..

[29] Buszewski B., Bukowska M., Ligor M., Staneczko-Baranowska I. (2019). A Holistic Study of Neonicotinoids Neuroactive Insecticides—Properties, Applications, Occurrence, and Analysis. Environ. Sci. Pollut. Res..

[30] Hassaan M.A., El Nemr A. (2020). Pesticides Pollution: Classifications, Human Health Impact, Extraction and Treatment Techniques. Egypt. J. Aquat. Res..

[31] Kardani F., Jelyani A.Z., Hashemi M., Rashedinia M., Shariati S., Mirzaei R., Mahdavinia M., Noori S.M.A. (2023). Determination of 323 Pesticide Residues in Iran’s Cereal by GC-MS and HPLC-UV Combined with QuEChERS Extraction and Mixed-Mode SPE Clean-up Method. J. Food Compos. Anal..

[32] Pany B.K., Sahu G., Pattnaik M., Pany K., Jena D., Kumar Pal A. (2020). Effect of Organochlorine Pesticides on Living Organisms and Environment Chemical Science Review and Letters Effect of Organochlorine Pesticides on Living Organisms and Environment. Chem. Sci. Rev. Lett..

[33] Madrigal J.M., Sargis R.M., Persky V., Turyk M.E. (2021). Multiple Organochlorine Pesticide Exposures and Measures of Sex Steroid Hormones in Adult Males: Cross-Sectional Findings from the 1999–2004 National Health and Nutrition Examination Survey. Int. J. Hyg. Environ. Health.

[34] Parada H., Sun X., Tse C.K., Engel L.S., Olshan A.F., Troester M.A. (2019). Plasma Levels of Dichlorodiphenyldichloroethene (DDE) and Dichlorodiphenyltrichloroethane (DDT) and Survival Following Breast Cancer in the Carolina Breast Cancer Study. Environ. Int..

[35] Ugalde-Resano R., Gamboa-Loira B., Mérida-Ortega Á., Rincón-Rubio A., Flores-Collado G., Piña-Pozas M., López-Carrillo L. (2023). Exposure to Organochlorine Pesticides and Female Breast Cancer Risk According to Molecular Receptors Expression: A Systematic Review and Meta-Analysis of Epidemiological Evidence. Curr. Environ. Health Rep..

[36] Freire C., Koifman R.J., Sarcinelli P., Rosa A.C., Clapauch R., Koifman S. (2012). Long Term Exposure to Organochlorine Pesticides and Thyroid Function in Children from Cidade Dos Meninos, Rio de Janeiro, Brazil. Environ. Res..

[37] Bandow N., Conrad A., Kolossa-Gehring M., Murawski A., Sawal G. (2020). Polychlorinated Biphenyls (PCB) and Organochlorine Pesticides (OCP) in Blood Plasma—Results of the German Environmental Survey for Children and Adolescents 2014–2017 (GerES V). Int. J. Hyg. Environ. Health.

[38] Rapini R., Marrazza G. (2016). Biosensor Potential in Pesticide Monitoring. Comprehensive Analytical Chemistry.

[39] Zhao W., Lu J., Lai Y., Hou Y., Zhao X., Wei Q., Zou X., Gou Z. (2023). Occurrences, Possible Sources, and Risk Impacts of Organochlorine Pesticides in Soil of Changchun Central Urban Area, Northeast China. Sustainability.

[40] Adeyinka G.C., Moodley B., Birungi G., Ndungu P. (2019). Evaluation of Organochlorinated Pesticide (OCP) Residues in Soil, Sediment and Water from the Msunduzi River in South Africa. Environ. Earth Sci..

[41] Zhu S., Zhou Y., Chao M., Zhang Y., Cheng W., Xu H., Zhang L., Tao Q., Da Q. (2024). Association between Organophosphorus Insecticides Exposure and Osteoarthritis in Patients with Arteriosclerotic Cardiovascular Disease. BMC Public Health.

[42] Mdeni N.L., Adeniji A.O., Okoh A.I., Okoh O.O. (2022). Analytical Evaluation of Carbamate and Organophosphate Pesticides in Human and Environmental Matrices: A Review. Molecules.

[43] Wu G., Shi W., Zheng L., Wang X., Tan Z., Xie E., Zhang D. (2024). Impacts of Organophosphate Pesticide Types and Concentrations on Aquatic Bacterial Communities and Carbon Cycling. J. Hazard Mater..

[44] Xie J., Li L., Khan I.M., Wang Z., Ma X. (2020). Flexible Paper-Based SERS Substrate Strategy for Rapid Detection of Methyl Parathion on the Surface of Fruit. Spectrochim. Acta A Mol. Biomol. Spectrosc..

[45] Sharma D., Wangoo N., Sharma R.K. (2021). Sensing Platform for Pico-Molar Level Detection of Ethyl Parathion Using Au–Ag Nanoclusters Based Enzymatic Strategy. Talanta.

[46] Colović M.B., Krstić D.Z., Lazarević-Pašti T.D., Bondžić A.M., Vasić V.M. (2013). Acetylcholinesterase Inhibitors: Pharmacology and Toxicology. Curr. Neuropharmacol..

[47] Zhang J., Guo J., Wu C., Qi X., Jiang S., Zhou T., Xiao H., Li W., Lu D., Feng C. (2020). Early-Life Carbamate Exposure and Intelligence Quotient of Seven-Year-Old Children. Environ. Int..

[48] Xie T., Huang J., Wu J., Zhang Q. (2024). Evaluation of Supercritical Fluid Chromatography Coupled to Tandem Mass Spectrometry for the Analysis of Pesticide Residues in Grain. J. Sep. Sci..

[49] Tomasevic A., Mijin D., Marinkovic A., Cvijetic I., Gasic S. (2019). Photocatalytic Degradation of Carbamate Insecticides: Effect of Different Parameters. Pestic. I Fitomedicina.

[50] Gu M., Chen S., Jiang J., Liao J., Long T., Xie Y. (2024). Do Pyrethroid Pesticide Residues in Chinese Mitten Crab Aquaculture Areas Have an Impact on the Ecological Environment?—A Case Study of Yangcheng Lake. J. Hazard Mater..

[51] Jia Q., Liao G., Chen L., Qian Y., Yan X., Qiu J. (2024). Pesticide Residues in Animal-Derived Food: Current State and Perspectives. Food Chem..

[52] Jeschke P., Nauen R. (2008). Neonicotinoids—From Zero to Hero in Insecticide Chemistry. Pest Manag. Sci..

[53] Ensley S.M. (2018). Neonicotinoids. Veterinary Toxicology.

[54] Fairbrother A., Purdy J., Anderson T., Fell R. (2014). Risks of Neonicotinoid Insecticides to Honeybees. Environ. Toxicol. Chem..

[55] Yari K., Rahmani A., Asgari G., Azarian Q., Bhatnagar A., Leili M. (2017). Degradation of Imidacloprid Pesticide in Aqueous Solution Using an Eco-Friendly Electrochemical Process. Desalination Water Treat..

[56] Malhotra N., Chen K.H.-C., Huang J.-C., Lai H.-T., Uapipatanakul B., Roldan M.J.M., Macabeo A.P.G., Ger T.-R., Hsiao C.-D. (2021). Physiological Effects of Neonicotinoid Insecticides on Non-Target Aquatic Animals—An Updated Review. Int. J. Mol. Sci..

[57] Basley K., Goulson D. (2018). Neonicotinoids Thiamethoxam and Clothianidin Adversely Affect the Colonisation of Invertebrate Populations in Aquatic Microcosms. Environ. Sci. Pollut. Res..

[58] Gautam P., Dubey S.K. (2023). Biodegradation of Neonicotinoids: Current Trends and Future Prospects. Curr. Pollut. Rep..

[59] Li Y., Li Y. (2024). Photodegradation of Neonicotinoid Insecticides Nitenpyram, Thiacloprid, and Acetamiprid in Water and Soil Environments. Bull. Environ. Contam. Toxicol..

[60] Zhao Y., Zhu Z., Xiao Q., Li Z., Jia X., Hu W., Liu K., Lu S. (2022). Urinary Neonicotinoid Insecticides in Children from South China: Concentrations, Profiles and Influencing Factors. Chemosphere.

[61] Wang P.-W., Huang Y.-F., Fang L.-J., Chen M.-L. (2024). Prenatal and Childhood Neonicotinoid Exposure and Neurodevelopment: A Study in a Young Taiwanese Cohort. Sci. Total Environ..

[62] D’Amore T., Smaoui S., Varzakas T. (2025). Chemical Food Safety in Europe Under the Spotlight: Principles, Regulatory Framework and Roadmap for Future Directions. Foods.

[63] Carrasco Cabrera L., Di Piazza G., Dujardin B., Marchese E., Medina Pastor P. (2025). The 2023 European Union Report on Pesticide Residues in Food. EFSA J..

[64] More S., Bampidis V., Benford D., Bragard C., Halldorsson T., Hougaard Bennekou S., Koutsoumanis K., Machera K., Naegeli H., Nielsen S. (2021). Statement on the Derivation of Health-Based Guidance Values (HBGVs) for Regulated Products That Are Also Nutrients. EFSA J..

[65] Brancato A., Brocca D., Ferreira L., Greco L., Jarrah S., Leuschner R., Medina P., Miron I., Nougadere A., Pedersen R. (2018). Use of EFSA Pesticide Residue Intake Model (EFSA PRIMo Revision 3). EFSA J..

[66] Damalas C.A., Eleftherohorinos I.G. (2011). Pesticide Exposure, Safety Issues, and Risk Assessment Indicators. Int. J. Environ. Res. Public Health.

[67] El-Sheikh E.S.A., Ramadan M.M., El-Sobki A.E., Shalaby A.A., McCoy M.R., Hamed I.A., Ashour M.B., Hammock B.D. (2022). Pesticide Residues in Vegetables and Fruits from Farmer Markets and Associated Dietary Risks. Molecules.

[68] Drabińska N., Marcinkowska M.A., Wieczorek M.N., Jeleń H.H. (2023). Application of Sorbent-Based Extraction Techniques in Food Analysis. Molecules.

[69] Ezati M., Moinfar S., Mohammadi S., Khayatian G. (2021). A Continuous Sample Drop Flow-Based Microextraction Method for Spectrophotometric Determination of Cobalt with 1-(2-Pyridylazo)-2-Naphthol in Water Samples. J. Anal. Chem..

[70] Kalogiouri N.P., Papadakis E.-N., Maggalou M.G., Karaoglanidis G.S., Samanidou V.F., Menkissoglu-Spiroudi U. (2021). Development of a Microwave-Assisted Extraction Protocol for the Simultaneous Determination of Mycotoxins and Pesticide Residues in Apples by LC-MS/MS. Appl. Sci..

[71] Zondo S., Mahlambi P. (2024). Comparison of Soxhlet and Microwave-assisted Extractions Efficiency for the Determination of Herbicides in Soil and Maize Crop: Cumulative and Health Risks Assessment. eFood.

[72] Barchańska H., Czalicka M., Giemza A. (2013). Simultaneous Determination of Selected Insecticides and Atrazine in Soil by Mae–GC–ECD. Arch. Environ. Prot..

[73] Tian Q., Li H., Chen L., Han B. (2024). Microwave-Assisted “One-Pot” Acidolysis and Extraction for the Rapid Determination of Mancozeb in Fruit and Vegetable Samples. J. Food Qual..

[74] Zhang H., Ren Y., Wei J., Ji Y., Bai X., Shao Y., Li H., Gao R., Wu Z., Peng Z. (2022). Optimization of the Efficient Extraction of Organic Components in Atmospheric Particulate Matter by Accelerated Solvent Extraction Technique and Its Application. Atmosphere.

[75] Chiesa L.M., Labella G.F., Panseri S., Britti D., Galbiati F., Villa R., Arioli F. (2017). Accelerated Solvent Extraction by Using an ‘in-Line’ Clean-up Approach for Multiresidue Analysis of Pesticides in Organic Honey. Food Addit. Contam. Part A.

[76] Horvat T., Jakovljević I., Sever Štrukil Z., Pehnec G. (2024). Optimisation of ASE for Determination of Organic Compounds Bound to Particulate Matter. Kem. U Ind..

[77] Morariu I.D., Avasilcai L., Cioanca O., Morariu B.-A., Vieriu M., Tanase C. (2022). The Effects of Honey Sulfonamides on Immunological and Hematological Parameters in Wistar Rats. Medicina.

[78] Morariu I.D. (2019). Immunochemical Assay of Chloramphenicol in Honey. Farmacia.

[79] Belmonte I.d.S., Pizzolato T.M., Gama M.R. (2022). Quaternary Ammonium Pesticides: A Review of Chromatography and Non-Chromatography Methods for Determination of Pesticide Residues in Water Samples. Trends Environ. Anal. Chem..

[80] Avellar Á., Prates G., Alves L., Prestes O., Adaime M., Zanella R. (2024). Multiresidue and Multiclass Determination of Current-Use Pesticides (Cups) in Water Samples by SPE and UHPLC-MS/MS. Quim Nova.

[81] Lee H., Cho Y., Jung G., Kim H., Jeong W. (2023). Comparison of Recovery Efficiency and Matrix Effect Reduction in Pesticide Residue Analysis: QuEChERS with d-SPE, SPE, and FaPEx in Apples and Korean Cabbage. Anal. Methods.

[82] Yun D.Y., Bae J.Y., Kang Y.J., Lim C.U., Jang G.H., Eom M.O., Choe W.J. (2024). Simultaneous Analysis of 272 Pesticides in Agricultural Products by the QuEChERS Method and Gas Chromatography with Tandem Mass Spectrometry. Molecules.

[83] Zgoła-Grześkowiak A., Grześkowiak T., Ligor M., Frankowski R. (2024). Achievements and Challenges of Matrix Solid-Phase Dispersion Usage in the Extraction of Plants and Food Samples. Processes.

[84] Zuin V.G., Yariwake J.H., Lanças F.M. (2003). Analysis of Pesticide Residues in Brazilian Medicinal Plants: Matrix Solid Phase Dispersion versus Conventional (European Pharmacopoeia) Methods. J. Braz. Chem. Soc..

[85] Souza M.R.R., Jesus R.A., Costa J.A.S., Barreto A.S., Navickiene S., Mesquita M.E. (2021). Applicability of Metal–Organic Framework Materials in the Evaluation of Pesticide Residues in Egg Samples of Chicken (Gallus Gallus Domesticus). J. Fur Verbraucherschutz Und Leb..

[86] Kemmerich M., Demarco M., Bernardi G., Prestes O.D., Adaime M.B., Zanella R. (2020). Balls-in-Tube Matrix Solid Phase Dispersion (BiT-MSPD): An Innovative and Simplified Technique for Multiresidue Determination of Pesticides in Fruit Samples. J. Chromatogr. A.

[87] Nolvachai Y., Amaral M.S.S., Herron R., Marriott P.J. (2023). Solid Phase Microextraction for Quantitative Analysis—Expectations beyond Design?. Green Anal. Chem..

[88] Agatonovic-Kustrin S., Gegechkori V., Kobakhidze T., Morton D. (2023). Solid-Phase Microextraction Techniques and Application in Food and Horticultural Crops. Molecules.

[89] Lancioni C., Castells C., Candal R., Tascon M. (2022). Headspace Solid-Phase Microextraction: Fundamentals and Recent Advances. Adv. Sample Prep..

[90] Rosendo L.M., Brinca A.T., Pires B., Catarro G., Rosado T., Guiné R.P.F., Araújo A.R.T.S., Anjos O., Gallardo E. (2023). Miniaturized Solid Phase Extraction Techniques Applied to Natural Products. Processes.

[91] Duan Y., Huang Y., Huang L., Xiang Z., Liu J., Liu S., Chen Z. (2024). In Vivo Tracing of Triazole Pesticides in Chinese Cabbage via a Novel Solid-Phase Microextraction Fiber. Food Control.

[92] Li W., Zhang J., Wang S., Ma Z., Feng J., Pei H., Liu Y. (2022). Simultaneous Determination of Three Herbicide Residues in Wheat Flour Based on the Hollow Fiber Supported Carbon Dots. J. Food Compos. Anal..

[93] Mungwari C.P., King’ondu C.K., Sigauke P., Obadele B.A. (2025). Conventional and Modern Techniques for Bioactive Compounds Recovery from Plants: Review. Sci. Afr..

[94] Edo G.I., Nwachukwu S.C., Ali A.B.M., Yousif E., Jikah A.N., Zainulabdeen K., Ekokotu H.A., Isoje E.F., Igbuku U.A., Opiti R.A. (2025). A Review on the Composition, Extraction and Applications of Phenolic Compounds. Ecol. Front..

[95] Baldino L., Scognamiglio M., Reverchon E. (2025). Green and Selective Supercritical Fluid Extraction of Essential Oil and Cannabidiol from *Cannabis sativa* L. Can. J. Chem. Eng..

[96] Yousefi M., Rahimi-Nasrabadi M., Mirsadeghi S., Pourmortazavi S.M. (2020). Supercritical Fluid Extraction of Pesticides and Insecticides from Food Samples and Plant Materials. Crit. Rev. Anal. Chem..

[97] Morariu I.D., Avasilcai L., Vieriu M., Panainte A.D., Bibire N. (2017). Novel Multiresidue Method for the Determination of Eight Trichothecene Mycotoxins in Pollen Samples Using QuEChERS-Based GC-MS/MS. Rev. De Chim..

[98] Abdulra’uf L.B., Junaid A.M., Lawal A.R., Ibrahim H.B., Tan G.H. (2025). Determination of Pesticide Residues in Beans Using QuEChERS Technique Coupled to Gas Chromatography-Mass Spectrometry: Multivariate Optimization of CEN and AOAC Methods. Food Chem..

[99] Tasic A.M., Ninković M., Pavlović I. (2024). Validation and Application of a Method for Determination of Multi-Class Pesticides in Muscle Chicken Breast Fillets Using QuEChERS Extraction and GC/MS. J. Vet. Res..

[100] Wilkowska A., Biziuk M. (2011). Determination of Pesticide Residues in Food Matrices Using the QuEChERS Methodology. Food Chem..

[101] Ma X., Wang J., Wu Q., Wang C., Wang Z. (2014). Extraction of Carbamate Pesticides in Fruit Samples by Graphene Reinforced Hollow Fibre Liquid Microextraction Followed by High Performance Liquid Chromatographic Detection. Food Chem..

[102] Kaenjun T., Tangtreamjitmun N. (2025). Spectrophotometric Determination of O-Phenylphenol in Canned Drinks Using Three-Phase Hollow-Fiber Liquid Phase Microextraction. Food Chem..

[103] Madikizela L.M., Pakade V.E., Ncube S., Tutu H., Chimuka L. (2020). Application of Hollow Fibre-Liquid Phase Microextraction Technique for Isolation and Pre-Concentration of Pharmaceuticals in Water. Membranes.

[104] Moema D., Makwakwa T.A., Gebreyohannes B.E., Dube S., Nindi M.M. (2023). Hollow Fiber Liquid Phase Microextraction of Fluoroquinolones in Chicken Livers Followed by High Pressure Liquid Chromatography: Greenness Assessment Using National Environmental Methods Index Label (NEMI), Green Analytical Procedure Index (GAPI), Analytical GREEnness Metric (AGREE), and Eco Scale. J. Food Compos. Anal..

[105] Lee J., Lee H.K., Rasmussen K.E., Pedersen-Bjergaard S. (2008). Environmental and Bioanalytical Applications of Hollow Fiber Membrane Liquid-Phase Microextraction: A Review. Anal. Chim. Acta.

[106] Khan W.A., Arain M.B., Yamini Y., Shah N., Kazi T.G., Pedersen-Bjergaard S., Tajik M. (2020). Hollow Fiber-Based Liquid Phase Microextraction Followed by Analytical Instrumental Techniques for Quantitative Analysis of Heavy Metal Ions and Pharmaceuticals. J. Pharm. Anal..

[107] Petrarca M.H., Cunha S.C., Fernandes J.O. (2024). Determination of Pesticide Residues in Soybeans Using QuEChERS Followed by Deep Eutectic Solvent-Based DLLME Preconcentration Prior to Gas Chromatography-Mass Spectrometry Analysis. J. Chromatogr. A.

[108] Pourhossein M., Khadem M., Omidi F., Heravizadeh O.R., Shahtaheri S.J. (2023). Development of a Green Single Drop Microextraction Based on Deep Eutectic Solvent and HPLC-UV for Trace Residue Analysis of Three Frequent-Used Pesticides. Iran. J. Public Health.

[109] Wahab S., Muzammil K., Nasir N., Khan M.S., Ahmad M.F., Khalid M., Ahmad W., Dawria A., Reddy L.K.V., Busayli A.M. (2022). Advancement and New Trends in Analysis of Pesticide Residues in Food: A Comprehensive Review. Plants.

[110] Moinfar S., Jamil L.A., Sami H.Z. (2020). Determination of Organophosphorus Pesticides in Juice and Water by Modified Continuous Sample Drop Flow Microextraction Combined with Gas Chromatography–Mass Spectrometry. Food Anal. Methods.

[111] Mohammed A.M., Moinfar S. (2023). Determination of Trimethoprim in Milk, Water and Plasma Using Protein Precipitation Combined with Liquid Phase Microextraction Method. J. Food Compos. Anal..

[112] Moinfar S., Jamil L.A., Sami H.Z., Ataei S. (2021). An Innovative Continuous Sample Drop Flow Microextraction for GC–MS Determination of Pesticides in Grape Juice and Water Samples. J. Food Compos. Anal..

[113] Jamil L.A. (2020). Optimization of New Sample Preparation Technique for the Determination of Methadone and Codeine in Plasma Sample by GC-FID. J. Braz. Chem. Soc..

[114] Méndez-Barredo L.H., Monribot-Villanueva J.L., Bojórquez-Velázquez E., Elizalde-Contreras J.M., Guerrero-Analco J.A., Ruiz-May E. (2023). Comparative Evaluation of Different Extraction Methods for Identification and Quantification of Glyphosate in Fortified Corn Flour. J. Mex. Chem. Soc..

[115] Han W., Tang H., Zhao L., Li Y., Men D., Dong M., Han L., Wang W. (2025). A Modified Method for the Simultaneous Determination of Fluindapyr and Its Metabolites Residues in Vegetables and Fruits by SPE and UHPLC MS/MS. J. Food Compos. Anal..

[116] Bakhshizadeh Aghdam M., Farajzadeh M.A., Afshar Mogaddam M.R. (2021). Partially Carbonized Cellulose Filter Paper as a Green Adsorbent for the Extraction of Pesticides from Fruit Juices. J. Chromatogr. A.

[117] Yin Y., Fan C., Cheng L., Shan Y. (2025). Efficient and Sensitive Detection of Organophosphate Pesticides in Orange Juice Using Dispersed Solid-Phase Extraction Based on Amorphous UiO-66. J. Sep. Sci..

[118] Nazari Koloujeh M., Iranifam M., Fathi A.A., Farajzadeh M.A., Afshar Mogaddam M.R. (2025). Development of COF@MOF Nanocomposite-Based Dispersive Solid-Phase Microextraction for the Extraction of Pesticides from Strawberries. Food Anal. Methods.

[119] Mujahid M., Latif S., Ahmed M., Shehzadi W., Imran M., Ahmad M., Asari A., Jehangir M., Mahmud Z. (2022). Modified Matrix Solid Phase Dispersion-HPLC Method for Determination of Pesticide Residue in Vegetables and Their Impact on Human Health: A Risk Assessment. Front. Chem..

[120] Yin R., Gao L., Qin D., Chen L., Niu N. (2022). Preparation of Porous Carbon-Based Molecularly Imprinted Polymers for Separation of Triazine Herbicides in Corn. Microchim. Acta.

[121] Fang Y., Wang W., Xu Y., Chen Q., Jiao T., Wei J., Chen Q., Chen X. (2025). Development of a Hydrophilic-Lipophilic-Balanced Copolymer@zirconium-Based Metal-Organic Framework-Based Solid-Phase Microextraction Probe for the Trace Determination of Organophosphorus Pesticides in Tea Infusions. Talanta.

[122] Pang Y., Zang X., Li H., Liu J., Chang Q., Zhang S., Wang C., Wang Z. (2020). Solid-Phase Microextraction of Organophosphorous Pesticides from Food Samples with a Nitrogen-Doped Porous Carbon Derived from g-C3N4 Templated MOF as the Fiber Coating. J. Hazard Mater..

[123] Delińska K., Yavir K., Kloskowski A. (2022). Head-Space SPME for the Analysis of Organophosphorus Insecticides by Novel Silica IL-Based Fibers in Real Samples. Molecules.

[124] Nakamura K., Otake T., Hanari N. (2023). Quantitative Determination of Organophosphorus, Pyrethroid, and Dithiolane Pesticide Residues in Brown Rice Using Supercritical Fluid Extraction and Liquid Chromatography–Tandem Mass Spectrometry. J. AOAC Int..

[125] Nakamura K., Otake T., Hanari N. (2020). Evaluation of Supercritical Fluid Extraction for the Determination of Neonicotinoid Pesticides in Green Onion. J. Environ. Sci. Health Part B.

[126] Cho S.M., Lee H.S., Park J.S., Lee S.J., Shin H.S., Chung Y.M., Choi H.N., Jung Y.H., Oh J.H., Yun S.S. (2021). Determination of Residual Triflumezopyrim Insecticide in Agricultural Products through a Modified QuEChERS Method. Foods.

[127] Muiambo S.L., Chaúque E.F.C., Gulamussen N.J., Chimuka L., Morifi E., Nyambe I. (2023). Modified QuEChERS Method for the Extraction and Quantification of Persistent Organic Compounds in Vegetables from Mozambican Local Markets. J. Hazard. Mater. Adv..

[128] Abasalizadeh A., Sorouraddin S.M., Farajzadeh M.A., Afshar Mogaddam M.R. (2022). Development of a Green Approach Based on DµSPE Combined with Deep Eutectic Solvent-Based DLLME for the Extraction of Some Pesticides from Vegetable Samples Prior to GC–FID and GC–MS. J. Iran. Chem. Soc..

[129] Szarka A., Búčiková K., Kostić I., Hrouzková S. (2020). Development of a Multiresidue QuEChERS–DLLME—Fast GC–MS Method for Determination of Selected Pesticides in Yogurt Samples. Food Anal. Methods.

[130] Raoufi A., Raoufi A.M., Ismailzadeh A., Soleimani Rad E., Kiaeefar A. (2023). Application of Hollow Fiber-Protected Liquid-Phase Microextraction Combined with GC-MS in Determining Endrin, Chlordane, and Dieldrin in Rice Samples. Environ. Geochem. Health.

[131] Jamil L.A., Sami H.Z., Aghaei A., Moinfar S., Ataei S. (2021). Combination of Modified Ultrasound-Assisted Extraction with Continuous Sample Drop Flow Microextraction for Determination of Pesticides in Vegetables and Fruits. Microchem. J..

[132] Sassolas A., Prieto-Simón B., Marty J.-L. (2012). Biosensors for Pesticide Detection: New Trends. Am. J. Anal. Chem..

[133] Hernández-Mesa M., Moreno-González D. (2022). Current Role of Mass Spectrometry in the Determination of Pesticide Residues in Food. Separations.

[134] Ciscato C.H.P., Bertoni Gebara A., Henrique Monteiro S. (2009). Pesticide Residue Monitoring of Brazilian Fruit for Export 2006–2007. Food Addit. Contam. Part B.

[135] Mutengwe M.T., Chidamba L., Korsten L. (2016). Pesticide Residue Monitoring on South African Fresh Produce Exported over a 6-Year Period. J. Food Prot..

[136] John P.J., Bakore N., Bhatnagar P. (2001). Assessment of Organochlorine Pesticide Residue Levels in Dairy Milk and Buffalo Milk from Jaipur City, Rajasthan, India. Environ. Int..

[137] Ohoro C.R., Wepener V. (2023). Review of Scientific Literature on Available Methods of Assessing Organochlorine Pesticides in the Environment. Heliyon.

[138] Prasad S.K., Kalpana D. (2024). Automation in Analytical Chemistry: The Role of AI in Chromatography. Int. J. Appl. Pharm..

[139] Singh Y.R., Shah D.B., Kulkarni M., Patel S.R., Maheshwari D.G., Shah J.S., Shah S. (2023). Current Trends in Chromatographic Prediction Using Artificial Intelligence and Machine Learning. Anal. Methods.

[140] Sinha N., Ray S. (2024). Application of Carbon Quantum Dots Derived from Waste Tea for the Detection of Pesticides in Tea: A Novel Biosensor Approach. ACS Omega.

[141] Sindhu S., Manickavasagan A. (2023). Nondestructive Testing Methods for Pesticide Residue in Food Commodities: A Review. Compr. Rev. Food Sci. Food Saf..

[142] Keklik M., Golge O., González-Curbelo M.Á., Kabak B. (2024). Determination of Pesticide Residues in Vine Leaves Using the QuEChERS Method and Liquid Chromatography-Tandem Mass Spectrometry. Foods.

[143] Gormez E., Golge O., González-Curbelo M.Á., Kabak B. (2023). Pesticide Residues in Mandarins: Three-Year Monitoring Results. Molecules.

[144] Penelope Mabunda K., Rejoice Maseko B., Ncube S. (2024). Development and Application of a New QuEChERS-Molecularly Imprinted Solid Phase Extraction (QuEChERS-MISPE) Technique for Analysis of DDT and Its Derivatives in Vegetables. Food Chem..

[145] Teixeira A.M., De Queiroz M.E.L.R., Rodrigues A.A.Z., de Oliveira A.F., Libardi V.M., de Freitas J.F. (2024). Determination of Pesticide Residues in Oat Flour Using Low-Temperature Partition Extraction and GC–MS Analysis. J. Food Sci. Technol..

[146] Wang Z., Jiang B., Pang C., Liu L., Zhou Q. (2024). Determination of Multiple Pesticide Residues and Dietary Intake Risk Assessment of 35 Pesticides in Beet from Five Provinces of Northern China. Sugar Tech..

[147] Morariu I.-D., Schiriac E.-C., Matiut D., Cuciureanu R. (2012). Method Validation for Simultaneous Determination of 12 Sulfonamides in Honey Using Biochip Array Technology. Farmacia.

[148] Bessaire T., Savoy M.-C., Ernest M., Christinat N., Badoud F., Desmarchelier A., Carrères B., Chan W.-C., Wang X., Delatour T. (2024). Enhanced Surveillance of >1100 Pesticides and Natural Toxins in Food: Harnessing the Capabilities of LC-HRMS for Reliable Identification and Quantification. Foods.

[149] Malm L., Liigand J., Aalizadeh R., Alygizakis N., Ng K., Fro̷kjær E.E., Nanusha M.Y., Hansen M., Plassmann M., Bieber S. (2024). Quantification Approaches in Non-Target LC/ESI/HRMS Analysis: An Interlaboratory Comparison. Anal. Chem..

[150] Chen Z., Daka Z., Yao L., Dong J., Zhang Y., Li P., Zhang K., Ji S. (2025). Recent Progress in the Application of Chromatography-Coupled Mass-Spectrometry in the Analysis of Contaminants in Food Products. Food Chem. X.

[151] Goh M.S., Lam S.D., Yang Y., Naqiuddin M., Addis S.N.K., Yong W.T.L., Luang-In V., Sonne C., Ma N.L. (2021). Omics Technologies Used in Pesticide Residue Detection and Mitigation in Crop. J. Hazard Mater..

[152] Shi Y., Jin H.-F., Ma X.-R., Cao J. (2024). Highly Sensitive Determination of Multiple Pesticide Residues in Foods by Supercritical Fluid Chromatography Coupled with Ion Mobility Quadrupole Time-of-Flight Mass Spectrometry. Food Res. Int..

[153] Bauer A., Luetjohann J., Rohn S., Jantzen E., Kuballa J. (2018). Development of a Suspect Screening Strategy for Pesticide Metabolites in Fruit and Vegetables by UPLC-Q-Tof-MS. Food Anal. Methods.

[154] Donley N., Cox C., Bennett K., Temkin A.M., Andrews D.Q., Naidenko O.V. (2024). Forever Pesticides: A Growing Source of PFAS Contamination in the Environment. Environ. Health Perspect..

[155] Zahra Z., Song M., Habib Z., Ikram S. (2025). Advances in Per- and Polyfluoroalkyl Substances (PFAS) Detection and Removal Techniques from Drinking Water, Their Limitations, and Future Outlooks. Emerg. Contam..

[156] Almeida M.O., Oloris S.C.S., Faria V.H.F., Ribeiro M.C.M., Cantini D.M., Soto-Blanco B. (2020). Optimization of Method for Pesticide Detection in Honey by Using Liquid and Gas Chromatography Coupled with Mass Spectrometric Detection. Foods.

[157] Elmi M., Ghane T., Daraei B., Eskandari S., Mohammadpour A., Amirahmadi M., Mousavi Khaneghah A. (2024). Monitoring of Pesticide Residue in Pistachio Nut Samples by LC/MS-MS. Food Chem..

[158] Wu X., Li J., Wei J., Tong K., Xie Y., Chang Q., Yu X., Li B., Lu M., Fan C. (2025). Multi-Residue Analytical Method Development and Dietary Exposure Risk Assessment of 345 Pesticides in Mango by LC-Q-TOF/MS. Food Control.

[159] Phichitsaenyakorn H., Bunkoed O. (2024). A Porous Composite Sorbent Incorporating a Metal Organic Framework and Zinc Oxide in Cryogel to Extract Carbamate Pesticides. Microchem. J..

[160] Mohamadi S., Akbari-adergani B., Sadighara P., Jannat B., Abdoli N., Mirzaei G., Zeinali T. (2023). Quantitative Analysis of Multiclass Pesticide Residues in Spinach, Iran. Appl. Food Res..

[161] Chen G., Shi L., Wang J., Zhu S., Sheng J., Yang X., Xu H. (2023). Pesticide Residues in Rice Planted in South and Southwest China. Food Addit. Contam. Part B.

[162] Hammood M.K., Arif M.A. (2024). Optimization and Validation of a GC-FID/QuEChERS Method for Quantitative Determination of Spiromesifen Residues in Tomato Fruits, Leaves and Soil Matrices. Anal. Bioanal. Chem. Res..

[163] Lobato A., Fernandes V.C., Pacheco J.G., Delerue-Matos C., Gonçalves L.M. (2021). Organochlorine Pesticide Analysis in Milk by Gas-Diffusion Microextraction with Gas Chromatography-Electron Capture Detection and Confirmation by Mass Spectrometry. J. Chromatogr. A.

[164] Yang K., Wang J., Gao S., Wei J., Chen T. (2025). The Determination Methods of Pesticide Multi-Residues and the Degradation Dynamics in Bitter Melon and Cucumber. J. Food Sci. Technol..

[165] Ramadan M.F.A., Abdel-Hamid M.M.A., Altorgoman M.M.F., AlGaramah H.A., Alawi M.A., Shati A.A., Shweeta H.A., Awwad N.S. (2020). Evaluation of Pesticide Residues in Vegetables from the Asir Region, Saudi Arabia. Molecules.

[166] Ramadevi R., Ramachandraiah C., Reddy G.V.S. (2023). Development of a Multi Residue Method for the Quantification of 45 Pesticides Using Gc-Ms/Ms and Study of Peeling Effect on Pesticide Residues in Citrus Fruits. Orient. J. Chem..

[167] Kim M., Cho M., Kim S.-H., Lee Y., Jo M.-R., Moon Y.-S., Im M.-H. (2024). Monitoring and Risk Assessment of Pesticide Residues in Fishery Products Using GC–MS/MS in South Korea. Toxics.

[168] Zheng K., Zheng H., Yu Y., Su J., Chen L., Zheng M., Liu L., Wu X., Chen D., Meng X. (2024). Simultaneous Determination of Four Pesticides Residues in Rice by Modified QuEChERS Coupled with GC-MS/MS. J. Food Compos. Anal..

[169] Bustamante C.M., Bravo N., Ruiz P., Grimalt J.O., Garí M. (2024). Method Optimization for a Simultaneous Determination of Neonicotinoid, Carbamate/Thiocarbamate, Triazole, Organophosphate and Pyrethroid Pesticides and Their Metabolites in Urine Using UPLC-MS/MS. J. Chromatogr. A.

[170] Elgueta S., Valenzuela M., Fuentes M., Ulloa P., Ramos C., Correa A., Molinett S. (2021). Analysis of Multi-Pesticide Residues and Dietary Risk Assessment in Fresh Tomatoes (*Lycopersicum esculentum*) from Local Supermarkets of the Metropolitan Region, Chile. Toxics.

[171] Crocoli L.C., Ramires N., Moura S. (2023). Determination of Pesticide Residues in Grapes Consumed in Natura and for Juice and Wine Production by High-Performance Liquid Chromatography with High Resolution Mass Spectrometry (HPLC-HRMS). Anal. Lett..

[172] Zhang Y., Li Z., Jiao B., Zhao Q., Wang C., Cui Y., He Y., Li J. (2023). Determination, Quality, and Health Assessment of Pesticide Residues in Kumquat in China. Foods.

[173] Wang B., Shi L., Ren P., Qin S., Li J., Cao J. (2024). Dissipation and Dietary Risk Assessment of the Fungicide Pyraclostrobin in Apples Using Ultra-High Performance Liquid Chromatography–Mass Spectrometry. Molecules.

[174] Morsi R., Ghoudi K., Meetani M.A. (2024). Determination and Health Risk Assessment of Carbamate Pesticide Residues in Date Palm Fruits (*Phoenix dactylifera*) Using QuEChERS Method and UHPLC-MS/MS. Sci. Rep..

[175] Diao Z., Di S., Qi P., Liu Z., Wang Z., Zhao H., Wang M., Zhang C., Wang X. (2024). Stereoselective Study on Chiral Fungicide Metconazole in Four Kinds of Fruits: Absolute Configuration, SFC-MS/MS Enantioseparation, Degradation and Risk Assessment. Food Chem..

[176] Zušťáková V., Dušek M., Jandovská V., Olšovská J. (2023). Screening and Quantification of Pesticide Residues in Ciders by Liquid Chromatography-High Resolution Mass Spectrometry. Czech J. Food Sci..

[177] Wang M., Wang C., Han X. (2017). Selection of Internal Standards for Accurate Quantification of Complex Lipid Species in Biological Extracts by Electrospray Ionization Mass Spectrometry—What, How and Why?. Mass Spectrom. Rev..

[178] Rimayi C., Odusanya D., Mtunzi F., Tsoka S. (2015). Alternative Calibration Techniques for Counteracting the Matrix Effects in GC–MS-SPE Pesticide Residue Analysis—A Statistical Approach. Chemosphere.

[179] Mirres A.C.d.M., Silva B.E.P.d.M.d., Tessaro L., Galvan D., de Andrade J.C., Aquino A., Joshi N., Conte-Junior C.A. (2022). Recent Advances in Nanomaterial-Based Biosensors for Pesticide Detection in Foods. Biosensors.

[180] Xu L., Abd El-Aty A.M., Eun J.B., Shim J.H., Zhao J., Lei X., Gao S., She Y., Jin F., Wang J. (2022). Recent Advances in Rapid Detection Techniques for Pesticide Residue: A Review. J. Agric. Food Chem..

[181] Dong J., Yang H., Li Y., Liu A., Wei W., Liu S. (2020). Fluorescence Sensor for Organophosphorus Pesticide Detection Based on the Alkaline Phosphatase-Triggered Reaction. Anal. Chim. Acta.

[182] Zhao G., Zhou B., Wang X., Shen J., Zhao B. (2021). Detection of Organophosphorus Pesticides by Nanogold/Mercaptomethamidophos Multi-Residue Electrochemical Biosensor. Food Chem..

[183] Liu S., Zheng Z., Li X. (2013). Advances in Pesticide Biosensors: Current Status, Challenges, and Future Perspectives. Anal. Bioanal. Chem..

[184] Sekhwama M., Mpofu K., Sivarasu S., Mthunzi-Kufa P. (2024). Applications of Microfluidics in Biosensing. Discov. Appl. Sci..

[185] Kant T., Shrivas K., Tejwani A., Tandey K., Sharma A., Gupta S. (2023). Progress in the Design of Portable Colorimetric Chemical Sensing Devices. Nanoscale.

[186] Wu H., Chen J., Yang Y., Yu W., Chen Y., Lin P., Liang K. (2022). Smartphone-Coupled Three-Layered Paper-Based Microfluidic Chips Demonstrating Stereoscopic Capillary-Driven Fluid Transport towards Colorimetric Detection of Pesticides. Anal. Bioanal. Chem..

[187] Peng S., Wang A., Lian Y., Zhang X., Zeng B., Chen Q., Yang H., Li J., Li L., Dan J. (2021). Smartphone-Based Molecularly Imprinted Sensors for Rapid Detection of Thiamethoxam Residues and Applications. PLoS ONE.

[188] Akdag A., Işık M., Göktaş H. (2021). Conducting Polymer-Based Electrochemical Biosensor for the Detection of Acetylthiocholine and Pesticide via Acetylcholinesterase. Biotechnol. Appl. Biochem..

[189] Hu H., Wang B., Li Y., Wang P., Yang L. (2020). Acetylcholinesterase Sensor with Patterned Structure for Detecting Organophosphorus Pesticides Based on Titanium Dioxide Sol-gel Carrier. Electroanalysis.

[190] Zhang X., Chen Y., Ling J., Li J. (2025). An Electrochemiluminescence Biosensor for Detection of Methyl Parathion with a Novel Probe Ru-MIL-100 via the Acetylcholinesterase Inhibition Coupled with the Reaction Between HAc and Triethylamine. Luminescence.

[191] Zhu J., Yin X., Zhang W., Chen M., Feng D., Zhao Y., Zhu Y. (2023). Simultaneous and Sensitive Detection of Three Pesticides Using a Functional Poly (Sulfobetaine Methacrylate)-Coated Paper-Based Colorimetric Sensor. Biosensors.

[192] Chen G., Jin M., Yan M., Cui X., Wang Y., Zheng W., Qin G., Zhang Y., Li M., Liao Y. (2019). Colorimetric Bio-Barcode Immunoassay for Parathion Based on Amplification by Using Platinum Nanoparticles Acting as a Nanozyme. Microchim. Acta.

[193] Lu Y., Tan Y., Xiao Y., Li Z., Sheng E., Dai Z. (2021). A Silver@gold Nanoparticle Tetrahedron Biosensor for Multiple Pesticides Detection Based on Surface-Enhanced Raman Scattering. Talanta.

[194] Arsawiset S., Sansenya S., Teepoo S. (2023). Nanozymes Paper−based Analytical Device for the Detection of Organophosphate Pesticides in Fruits and Vegetables. Anal. Chim. Acta.

[195] Qin J., Chen Y., Jia L., Chang T., Chen J., Li Y., Ma Z., Zhan Y., Yang H. (2025). An Electrochemical Biosensor with Perovskite/AuNPs Composite for Sensitive Determination of Fenitrothion. Microchem. J..

[196] Tessaro L., Aquino A., de Almeida Rodrigues P., Joshi N., Ferrari R.G., Conte-Junior C.A. (2022). Nucleic Acid-Based Nanobiosensor (NAB) Used for Salmonella Detection in Foods: A Systematic Review. Nanomaterials.

[197] El Alami A., Lagarde F., Tamer U., Baitoul M., Daniel P. (2016). Enhanced Raman Spectroscopy Coupled to Chemometrics for Identification and Quantification of Acetylcholinesterase Inhibitors. Vib. Spectrosc..

[198] Nie Y., Teng Y., Li P., Liu W., Shi Q., Zhang Y. (2018). Label-Free Aptamer-Based Sensor for Specific Detection of Malathion Residues by Surface-Enhanced Raman Scattering. Spectrochim. Acta A Mol. Biomol. Spectrosc..

[199] Xu M.-L., Gao Y., Han X.X., Zhao B. (2017). Detection of Pesticide Residues in Food Using Surface-Enhanced Raman Spectroscopy: A Review. J. Agric. Food Chem..

[200] Chen H., An L., Li M., Liu H., Jin Z., Ma H., Ma J., Zhou J., Duan R., Zhang D. (2024). A Self-Assembled 3D Nanoflowers Based Nano-ELISA Platform for the Sensitive Detection of Pyridaben. Food Chem..

[201] Dávila E.L., Romero O.R., Du Laing G., Spanoghe P. (2021). Du Use of Enzyme-Linked ImmunoSorbent Assay Technique to Monitor Pesticide Residues in Horticultural Crops. J. Chem. Eng. Theor. Appl. Chem..

[202] López Dávila E., Houbraken M., Gil Unday Z., Romero Romero O., Du Laing G., Spanoghe P. (2020). ELISA, a Feasible Technique to Monitor Organophosphate, Carbamate, and Pyrethroid Residues in Local Vegetables. Cuban Case Study. SN Appl. Sci..

[203] Li H., He S., Liu G., Li C., Ma Z., Zhang X. (2021). Residue and Dissipation Kinetics of Toosendanin in Cabbage, Tobacco and Soil Using IC-ELISA Detection. Food Chem..

[204] Jia M., E Z., Zhai F., Bing X. (2020). Rapid Multi-Residue Detection Methods for Pesticides and Veterinary Drugs. Molecules.

